# Cascading climate effects in deep reservoirs: Full assessment of physical and biogeochemical dynamics under ensemble climate projections and ways towards adaptation

**DOI:** 10.1007/s13280-023-01950-0

**Published:** 2023-11-08

**Authors:** Chenxi Mi, Tom Shatwell, Xiangzhen Kong, Karsten Rinke

**Affiliations:** 1 Department of Lake Research, Helmholtz Centre for Environmental Research, Brückstraße 3A, 39114 Magdeburg, Germany; 2 College of Water Conservancy, Shenyang Agricultural University, Shenyang, China; 3 State Key Laboratory of Lake Science and Environment, Nanjing Institute of Geography and Limnology, Chinese Academy of Sciences, Nanjing, China

**Keywords:** Climate adaptive strategy, Global warming, Metalimnetic hypoxia, Stratification phenology, Water quality simulation

## Abstract

We coupled twenty-first century climate projections with a well-established water quality model to depict future ecological changes of Rappbode Reservoir, Germany. Our results document a chain of climate-driven effects propagating through the aquatic ecosystem and interfering with drinking water supply: intense climate warming (RCP8.5 scenario) will firstly trigger a strong increase in water temperatures, in turn leading to metalimnetic hypoxia, accelerating sediment nutrient release and finally boosting blooms of the cyanobacterium *Planktothrix rubescens*. Such adverse water quality developments will be suppressed under RCP2.6 and 6.0 indicating that mitigation of climate change is improving water security. Our results also suggested surface withdrawal can be an effective adaptation strategy to make the reservoir ecosystem more resilient to climate warming. The identified consequences from climate warming and adaptation strategies are relevant to many deep waters in the temperate zone, and the conclusion should provide important guidances for stakeholders to confront potential climate changes.

**Supplementary Information:**

The online version contains supplementary material available at 10.1007/s13280-023-01950-0.

## Introduction

In comparison to natural lakes, reservoirs are often characterized by more intense anthropogenic use and management, in particular those with multiple uses like water supply, hydropower and flood protection (Rinke et al. 2019). Among them, drinking water reservoirs are of outstanding importance for human health, as well as economic and social development. Their water quality is necessarily of high relevance (Wentzky et al. 2019), but reservoir water quality appears to be particularly sensitive to climate change (Mi et al. 2019). Atmospheric warming and wind stilling, for example, reduce mixing, intensify stratification, facilitate nutrient release and decrease oxygen concentration in deep waters (Woolway et al. 2020). At the ecosystem scale, these physical, biogeochemical and biological processes interact (North et al. 2014), so that warming, deoxygenation and nutrient dynamics further boost the emergence of cyanobacterial blooms, as has been observed in reservoirs worldwide (Ho et al. 2019).

The development of aquatic ecosystems under projected climate change is currently a relevant and critical concern at the global scale (Woolway et al. 2020), particularly because water warming is expected to be the key factor triggering water quality deterioration (Zhang et al. 2015). In this respect, it is astonishing to note that the majority of existing studies, which target impacts of projected climate change on lakes and reservoirs, have solely focussed on physical changes, that is, the increase in surface temperature, the alteration in mixing types or the occurrence of lake heatwaves (Woolway et al. 2022). By contrast, only few studies really address this topic at the ecosystem scale. Using a water quality model driven by meteorological output from a climate model (MRI-CGCM2) under the SRES-A2 scenario, Komatsu et al. (2007) showed that water temperature in Shimajigawa Reservoir, Japan, would strongly increase by 2091–2100 compared to historical conditions (1991–2001), promoting aerobic decomposition in the water column and an upward flux of phosphorus from sediments. Recently by coupling the climate model GFDL-ESM2M (under scenario RCP8.5) with the lake model MyLake-Sediment, Messina et al. (2020) projected water quality conditions for Lake Auburn, USA. Their results illustrated a strong increase of phosphorus concentration in the period 2046–2055 compared to 2006–2015. While such studies clearly showed that climate warming is accompanied by a eutrophication trend, they did not include management responses or potential climate adaptation strategies. This is partly due to the fact that previous studies applied only a very simple ecological model that either left out algal growth or used a simplified bulk algal model with only one algal group. However, when it comes to harmful algal blooms and sensitive human activities like bathing or drinking water production, the dynamics and composition of the phytoplankton are of strong interest. The solid projection of physical structure, biogeochemical cycling and ecological features from one coherent approach is currently a critical concern to understand whole-environment dynamics, and adaptive management strategies for lakes and reservoirs under future climate conditions.

Results from long-term observations (Ho et al. 2019) document strongly negative effects, drawn from historical climate warming, on the ecological status of lakes and reservoirs which will possibly continue over the twenty-first century (Råman Vinnå et al. 2021). Water managers, consequently, are calling for climate adaptation strategies and science-based management practices built on reliable future prediction of water quality. Among classical reservoirs management strategies, selective water withdrawal is supposed to be a fast-reaction one which is effective in manipulating the physical and ecological structure of targeted waters (Mi et al. 2019). The commonly used hypolimnetic withdrawal (Weber et al. 2017), for example, promotes heat transport into the deeper water column facilitating hypolimnetic oxygen consumption and nutrient release from sediments (Carr et al. 2019). The additional nutrients fuel growth of phytoplankton, initiating a cycle of eutrophication and further enhancing hypolimnetic oxygen demand. By comparison, application of surface withdrawal (SW) can strongly mitigate warming intensities and thus decelerate all temperature-dependent biochemical processes (Mi et al. 2019). Moreover, selective withdrawal can be used to effectively export water with bad quality while keeping the good quality water inside the reservoir (Zouabi-Aloui et al. 2015). Such findings give rise to the idea that reservoir managers can apply withdrawal strategies to buffer or even offset adverse effects from climate warming on reservoir ecosystems. However, this has never been explored systematically, i.e. by an integrated workflow of climate projections, lake physical and ecological modelling and a set of appropriate adaptation strategies applied to a real water body, which is the main topic we want to address in this study.

To determine climate-driven trajectories of reservoir ecosystems in the twenty-first century and mechanisms behind them, we applied a well-established water quality model (CE-QUAL-W2) to Rappbode Reservoir, Germany’s largest drinking water reservoir. Results from three future climate scenarios (i.e. RCP2.6, 6.0 and 8.5), driven by four General Circulation Models (GCMs), were used as climate boundary conditions. Moreover, we explored climate adaptation strategies in reservoir operation using selective water withdrawal. As a typical drinking water reservoir in the temperate zone, we believe the conclusion from our study reaches far beyond the single case of Rappbode Reservoir, and should inspire other stakeholders adapting their management methods to confront with potential climate changes.

## Methods

### Study site

Rappbode Reservoir, Germany’s largest drinking water reservoir (Fig. S1), is located within the Harz mountains, with a bottom elevation at 338 m above sea level (m a.s.l.) and dam crest at 423 m a.s.l. (Mi et al. 2020b). The total volume of the reservoir is around 1.1 × 10^8^ m^3^ and its mean (maximum) depth is 28.6 m (89 m). It receives water from two pre-reservoirs (Hassel and Rappbode) and a transfer tunnel from Konigshütte Reservoir. The dam has in total 6 outlets at elevations of 345, 360, 370, 380, 390 and 400 m a.s.l. (Fig. S1). The Rappbode Reservoir has two separate outflows: the downstream withdrawal, which flows into Wendefurth Reservoir (taken usually from the outlet at 345 m a.s.l.), and the raw water abstraction for the drinking water supply (mostly taken from the outlets at 360 m a.s.l and 370 m a.s.l, Mi et al. (2019)). Rappbode Reservoir is dimictic and fully mixes in spring and autumn, experiencing weak inverse stratification in most winters and strong stratification in summer. After substantial nutrient load reductions at 1990, the reservoir is nowadays a mesotrophic water with an annual average TP concentration of 0.02 mg L^−1^. Metalimnetic oxygen minima (hereafter MOM) regularly occur, as documented by long-term measurements (Wentzky et al. 2019).

### Reservoir model for simulating hydrodynamics and water quality

The water quality model, CE-QUAL-W2 (hereafter W2, version 4.1, http://www.ce.pdx.edu/w2/), is a two-dimensional (2D) model simulating the hydrodynamics and ecosystem dynamics along spatially resolved longitudinal-vertical axes (Cole and Wells 2006). The model grid includes 4 branches with 106 horizontal segments and a vertical resolution of 1 m (3876 grid cells in total), which fully represents the topographical structure of the reservoir (Fig. S2) as indicated by similar elevation–volume relationship drawn from generated and original lake basins.

The hydrodynamic (i.e. water temperature) and ecological modules [i.e. oxygen, nutrients and two phytoplankton groups representing diatoms and the cyanobacterium *Planktothrix rubescens* (hereafter *P. rubescens*)] provide the required level of complexity for a proper process-oriented inclusion of climate effects, biogeochemical processing and management measures. For more information about the phytoplankton groups defined in the model, readers are referred to the supplementary material. Other details of the model setup were described in Mi et al. (2020a).

### Model calibration and validation

#### Data collection

We calibrated (validated) the model for the year 2016 (2015) using high-quality temperature and water quality measurements. Input data for daily inflow discharge, inflow water temperatures and outflow discharge, and biweekly nutrients (including silicate, nitrate, phosphate and ammonia) were provided by the reservoir authority and our monitoring programme using a YSI-6200 probe and wet chemical analysis meeting German standards (Wentzky et al. 2019), respectively. For the meteorological inputs, air temperature, relative humidity, wind speed and direction at hourly resolution were drawn from our monitoring buoy deployed in the reservoir (see Mi et al. 2019). Due to the data gaps, shortwave radiation measurements before April 2015 were taken from the Harzgerode station of the German Meteorological Service. Cloud cover data were taken from the Harzgerode station since it was not measured on the buoy.

The field data, measured at the deepest point of reservoir at several depths (Fig. S1), were used to analyse model performance in both years with respect to water temperature and DO concentration (measured biweekly with a Hydrolab DS5 probe), nutrient concentration (for silicate and nitrate measured biweekly in 2016 and monthly in 2015, determined with the same method as for the inflows) and phytoplankton concentration (for *P. rubescens* and diatoms measured biweekly in 2016 and six times from June to October in 2015 with the unit of µg chl-*a* L^−1^, by a bbe Moldaenke Fluoroprobe). We did not include phosphate and ammonium in this performance analysis since their measured concentrations were always lower than the detection limit (phosphate: 0.003 mg L^−1^, ammonium: 0.001 mg L^−1^).

#### Calibration/validation methods

We firstly tuned the parameters for water temperature, and subsequently the ecological part (Sadeghian et al. 2018; Chuo et al. 2019). We used the traditional “trial and error” method during the process in which wind sheltering (WSC), shading (SHADE) and light extinction coefficients (EXH2O) were considered for the temperature calibration. More parameters were related to the ecological simulation, and a detailed description of the calibration process is provided in the supplementary material. We used coefficient of determination (*R*^2^), mean absolute error (MAE) and root-mean-square error (RMSE) to analyse model accuracy and performance. All calculations for pre- and post-processing were done using R packages ‘dplyr’ (v1.1.0, Wickham et al. 2023), ‘data.table’ (v1.14.6, Dowle and Srinivasan 2022) and ‘MASS’ (v7.3-58.1, Venables and Ripley 2013).

### Simulation protocol for future climate

Future climate projections were provided by the ISIMIP database (Inter-Sectoral Impact Model Intercomparison Project, see Hempel et al. 2013), for the grid cell containing Rappbode Reservoir (51.74°N, 10.89°E). The projections consist of low (RCP2.6), medium (RCP6.0) and high (RCP8.5) greenhouse gas emission scenarios propagated through four General Circulation Models (GCMs, i.e. IPSL-CM5A-LR, HadGEM2-ES, GFDL-ESM2M and MIROC-ESM-CHEM), and please refer to supplementary material for different performance by four GCMs in future climate projections. The GCM data have been bias corrected using the modified version of the trend-preserving method, and agree well with long-term observations worldwide (Casanueva et al. 2019). The performance of the ISIMIP data in reproducing the meteorological conditions at Rappbode Reservoir has been confirmed in Mi et al. (2020b). Four GCMs, in the database, project the climate from 2006 to 2099. Based on the ensemble means, the region around Rappbode Reservoir will warm by 0.05 °C decade^−1^ under RCP2.6, while the warming reaches to 0.2 °C decade^−1^ for RCP6.0 and further to 0.5 °C decade^−1^ for RCP8.5 (Martínez-Solanas et al. 2021).

We did not model future hydrological conditions since inflows and outflows in Rappbode Reservoir are highly regulated by reservoir managers (e.g. a bypass system at Königshütte Reservoir), which cannot be predicted just based on precipitation data. Instead we calculated daily average inflow and outflow discharge by fitting a generalized additive model (GAM) to long-term measurements (1996–2015). We then used the results as hydrological input to drive W2, which is similar to the approach by Ladwig et al. (2018). In the current management regime and in our reference scenarios, all outflow is withdrawn from the hypolimnion (i.e. from 345 m a.s.l. for downstream discharge, and 360 m a.s.l. and 370 m a.s.l. for drinking supply). More details about the model setup of future inflow temperature and nutrient concentration are given in the supplementary material.

### Adaptive management by alternative withdrawal strategies under future climate warming

To examine the effect of alternative withdrawal strategies on the reservoir ecosystem under climate warming, we defined adaptive management scenarios using other withdrawal depths, under the worst-case climate scenario RCP8.5, in which the downstream discharge was withdrawn from the surface (i.e. the highest outlet at 400 m a.s.l. instead of 345 m a.s.l.). The raw water for drinking supply was still withdrawn from the bottom two outlets (360 and 370 m a.s.l.) to meet the requirement for cold, algae-free water. All the other settings in this scenario were the same as the reference scenario described in “Simulation protocol for future climate” section.

According to the major water quality targets, we focussed on future changes of water temperature, DO concentration and the two phytoplankton groups. We calculated the buoyancy frequency (*N*^2^, see Fang and Stefan (2009)) to analyse long-term trend of stratification intensity driven by the climate projections:

1 $${N}^{2}=\frac{g}{\rho }\frac{\text{d}\rho }{\text{d}z},$$

where *g* refers to gravity acceleration, $\rho$ is density and *z* is depth. We further calculated stratification duration according to the water temperature projections, defined as total number of days when the temperature difference between the top and bottom layer is greater than 1 °C (Fang and Stefan 1999). Additionally, we quantified the volumetric oxygen consumption rate (VOC, including the oxygen consumption in both the sediment and the water column) under future climate projections, based on the simulation output. Moreover, the projected phosphate concentration was incorporated into the analysis because Rappbode Reservoir is a strongly P-limited system (see “Study site” section). All statistical analyses for the scenarios were performed using R version 3.3.2 (R Core Team 2016). For more details about the R packages used in this part, please see the supplementary material.

## Results

### Model performance during the calibration and validation period

#### Thermal structure

The model showed excellent performance in capturing thermal dynamics in Rappbode Reservoir for both the calibration (year 2016) and validation (year 2015) period, with an RMSE for water temperature of 0.45 °C and 0.65 °C, respectively, and *R*^2^ close to 1 (Table 1). The simulated vertical temperature, in terms of annual mean, was 6.06 °C in 2015, and 6.13 °C in 2016, which is only marginally lower than measurements (6.33 °C and 6.37 °C for the 2 years) indicating a low model bias. The observed stratification phenology in the reservoir (e.g. stratification onset/offset, changes of thermocline depth and hypolimnetic volume, etc.) was also well reproduced by the model (Fig. S3).

**Table 1:** Calibration and validation results for water quality variables (*DO* dissolved oxygen, *RMSE* root mean squared error, *R*^2^ coefficient of determination, *MAE* mean absolute error)

	Calibration			Validation		
	*R*^2^	RMSE	MAE	*R*^2^	RMSE	MAE
Water temperature (°C)	0.99	0.45	0.21	0.97	0.65	0.48
Silicate (mg L^−1^)	0.84	0.29	0.13	0.65	0.31	0.17
Nitrate (mg L^−1^)	0.69	0.14	0.06	0.37	0.11	0.05
*P. rubescens* (µg chl-a L^−1^)	0.56	0.65	0.23	0.48	0.49	0.23
Diatom (µg chl-a L^−1^)	0.55	0.72	0.47	0.21	0.81	0.38
DO (mg L^−1^)	0.84	0.95	0.67	0.81	0.72	0.48

#### Phytoplankton

The model performed well in reproducing seasonal dynamics of the two phytoplankton groups during the calibration and validation period (Figs. S4–S7). The model captured both the observed growth of *P. rubescens* in the metalimnion (around 10 m depth) during late summer and the spring diatom bloom, with *R*^2^ approaching 0.6 and RMSE below 0.8 µg L^−1^ (Table 1). Although simulation results nicely reproduced the observed patterns in diatom distribution, they occasionally showed smaller differences in concentration, especially during the late autumn, in comparison to measurements.

***Nutrients***: The model satisfactorily captured the spatial–temporal dynamics of nutrients in the reservoir, which reproduced silicate better than nitrate (Table 1). For example, the *R*^2^ values for silicate were 0.84 (calibration) and 0.65 (validation), whereas for nitrate, they were 0.69 and 0.37. Due to algal nutrient uptake within the surface layers and mineralization at the sediment, observed nutrient concentrations in the bottom water were always higher than at the surface, and this pattern was accurately captured by the simulations (Figs. S8–S11). The model reproduced the spatial and temporal dynamics of the nutrient concentrations and gradients, but slightly underestimated nitrate and overestimated silicate concentrations (Figs. S8–S3).

#### Dissolved Oxygen

**Fig. 1:**
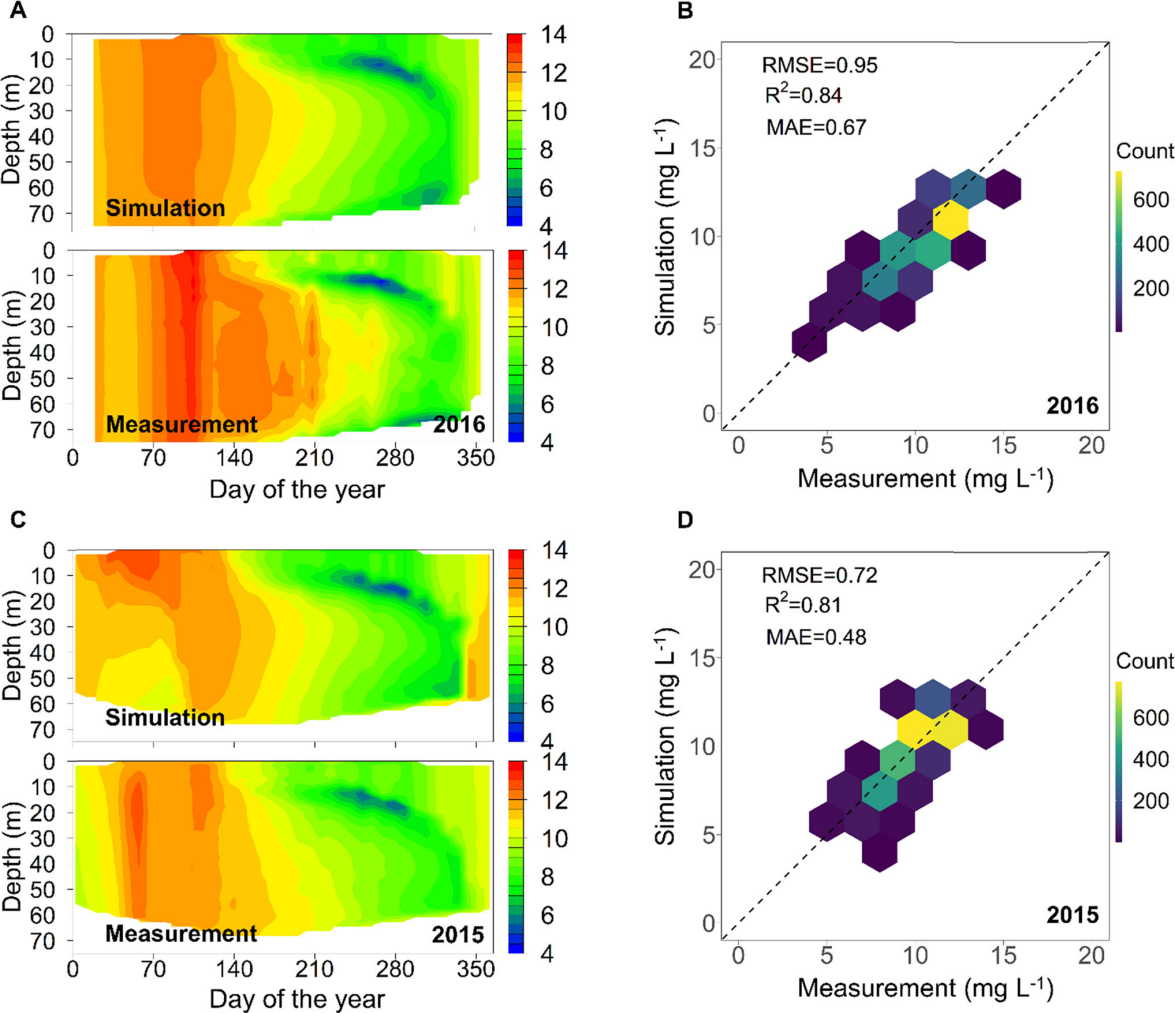
Comparison between simulated and measured DO concentration during the calibration (**A** and **B**) and validation (**C** and **D**) period. The colour scale, in subfigure B and D, denotes the amount of samples per hexagon, and the straight line has a slope of one with an intercept of zero (1:1 line). The number at bottom-right shows the year of comparison

Simulated DO also agreed well with the field data for both calibration and validation (see Table 1). According to the measurements, until spring mixis at around day 100, DO is typically homogeneously distributed in the vertical direction. Afterwards pronounced gradients develop during the stratification period, with local DO minima at the bottom and within the metalimnion (i.e. MOM shown in “Study site” section). The model accurately captured the patterns above, e.g. the appearance of the MOM in August with concentrations decreasing to 4 mg L^−1^ (also reflected in the measurements, Fig. 1), its decline starting in early October, and its total disappearance in winter (see Fig. 1).

### Response of aquatic ecosystems to projected climate change

Climate change significantly affected physical, chemical and biological variables in the reservoir, with strong contrasts between different RCP scenarios. Overall, our work documented a cascading response of the aquatic ecosystem in Rappbode Reservoir under intense climate warming (i.e. RCP8.5) starting with (i) rising water temperatures, (ii) stronger DO depletion approaching hypoxic/anoxic conditions in the metalimnion and hypolimnion, (iii) eutrophication due to intensifying internal nutrient loading and (iv) shifting plankton succession towards overwintering populations of the cyanobacterium *P. rubescens* and an overall increase of cyanobacterial dominance. This response, by comparison, is much weaker or not expressed under the other scenarios.

**Fig. 2:**
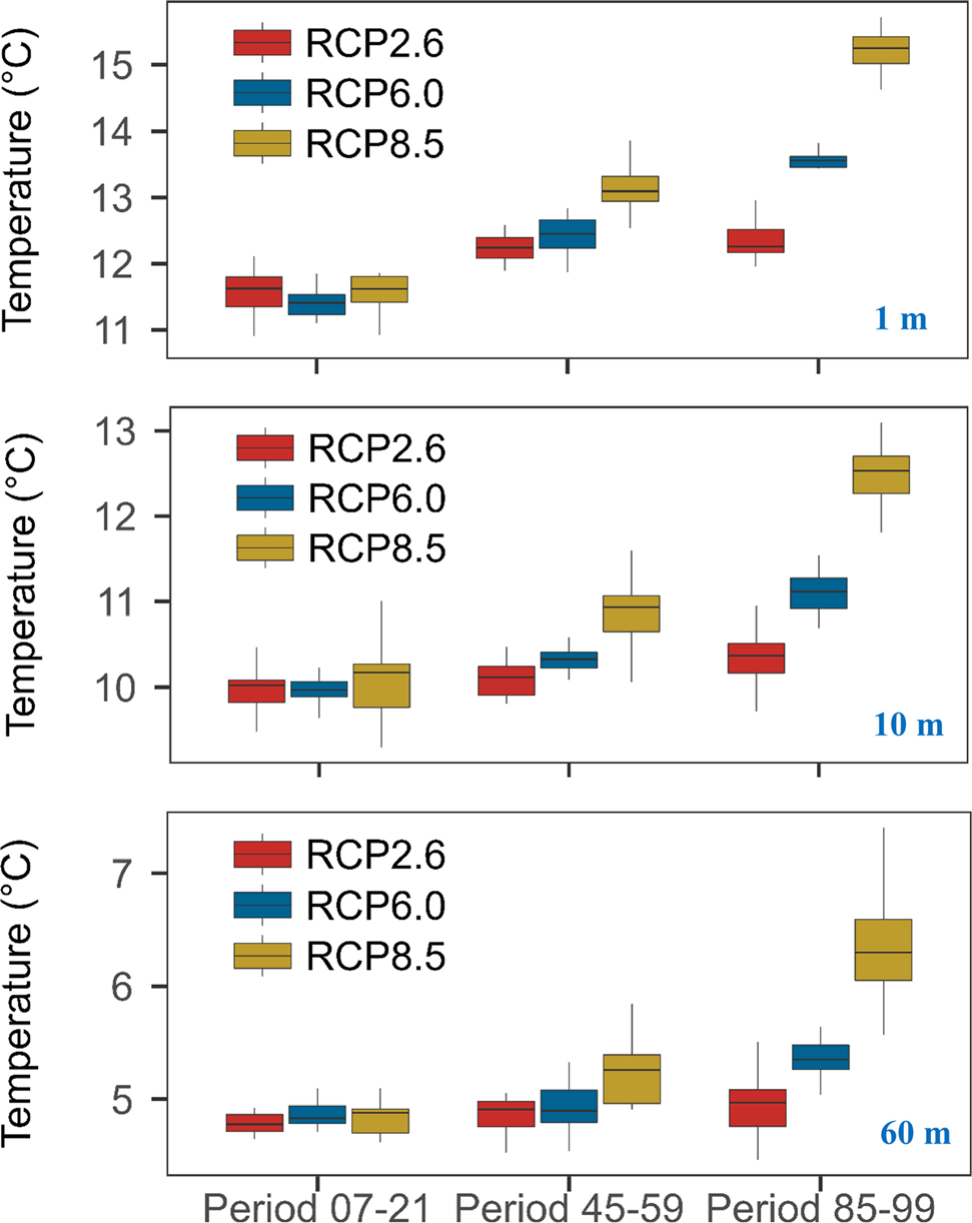
Projected mean and variability of water temperature, for Rappbode Reservoir, at the depth of 1 m (top), 10 m (middle) and 60 m (bottom) during 3 periods in the twenty-first century (Period 07–21: from 2007 to 2021, Period 45–59: from 2045 to 2059, Period 85–99: from 2085 to 2099) under RCP2.6, 6.0 and 8.5. The boxplots indicate the medians, upper (lower) quartiles and full ranges of the 15 annual ensemble mean temperatures for each period

Expectedly, simulations showed that water temperatures will undergo the strongest increase under RCP8.5, and the weakest increase under RCP2.6 (Fig. S12). For example, the median surface temperature under RCP2.6 (at 1 m depth), over all four GCMs, was 11.5 °C at the beginning of the twenty-first century (from 2007 to 2021, hereafter Period 07–21), 12.2 °C in the middle of the century (from 2045 to 2059, hereafter Period 45–59) and remained at this level (12.4 °C) until the end of the century (from 2085 to 2099, hereafter Period 85–99, see Fig. 2). Under RCP8.5, in comparison, the median surface temperature dramatically increased from 11.4 °C (Period 07–21) to 13.1 °C (Period 45–59) and finally to 15.2 °C in Period 85–99. Under RCP 6.0, the increase was intermediate, ranging from 11.5 °C to 12.4 °C and then to 13.3 °C in the three periods, respectively (Fig. 2). Similar temperature trajectories are also visible in the metalimnion (10 m depth) but bottom temperature dynamics (60 m depth) differed. The bottom water warmed substantially only under RCP8.5 with deep-water temperatures reaching 7 °C by the end of the century, presumably due to the increase in heat and mixing temperatures (see “Cascading effects of future climate projection on reservoir ecosystems” section). Such deep-water warming was only marginal in the other two scenarios (Fig. 2). Besides water temperatures, stratification intensity (represented by buoyancy frequency) also strongly increased under RCP8.5 (see below).

**Fig. 3:**
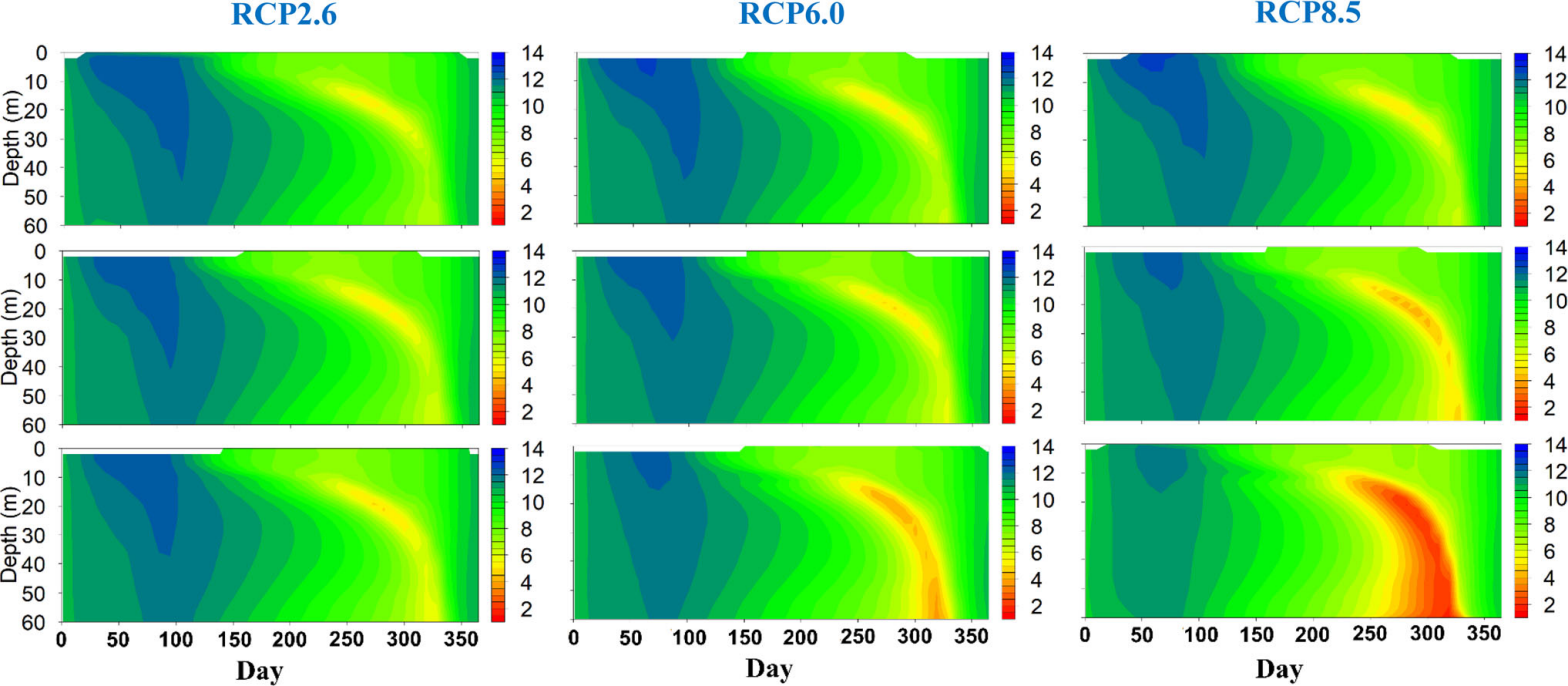
Future projections of DO concentration (mg L^−1^), for Rappbode Reservoir, under RCP2.6 (left), RCP6.0 (middle) and RCP8.5 (right). The top row indicates the ensemble average results, for every Julian day, in Period 07–21 (from 2007 to 2021), the middle row indicates the results in Period 45–59 (from 2045 to 2059) and the bottom row indicates the results in Period 85–99 (from 2085 to 2099)

**Fig. 4:**
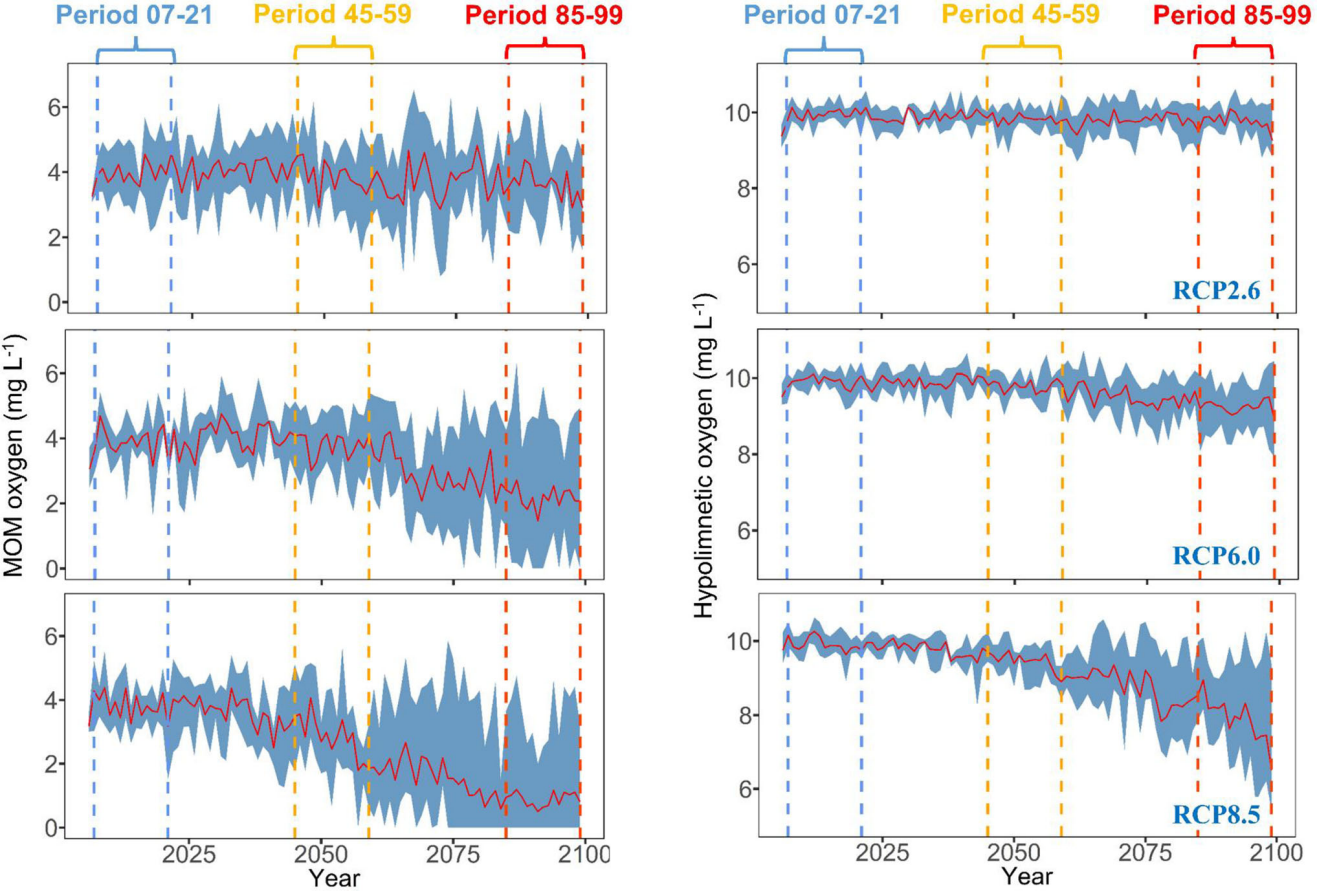
Future projections of the MOM (metalimnetic oxygen minima, left) and hypolimnetic (right) DO concentration, for Rappbode Reservoir, under RCP2.6 (upper), RCP6.0 (middle) and RCP8.5 (bottom). The red lines indicate the annual ensemble average results driven by four climate models, and the blue shaded areas indicate the annual minimum and maximum results from the ensemble. Dashed lines, in the vertical direction, indicate 3 periods in the twenty-first century

Climate warming strongly decreased DO concentration in the reservoir at a rate proportional to the increase in air temperature of each RCP (Figs. 3, 4). The concentration in surface layers (within 2 m depth) continually decreased over the century, with the lowest rate under RCP2.6 (− 0.01 mg L^−1^ decade^−1^, annual ensemble means) and highest under RCP8.5 (− 0.11 mg L^−1^ decade^−1^, Table 2). DO concentration in the MOM (i.e. the lowest DO concentration in the upper 35 m, following Mi et al. (2020a)) consistently decreased over time across all model ensembles. This decrease was weak under RCP2.6, strong under RCP6.0 and drastic under RCP8.5 (Table 2). The same pattern was projected for the hypolimnetic DO concentration, where the mean trend below 35 m was − 0.02 mg L^−1^ decade^−1^ under RCP 2.6, − 0.09 mg L^−1^ decade^−1^ under RCP 6.0 and − 0.22 mg L^−1^ decade^−1^ under RCP 8.5 (Table 2). Interestingly, the MOM concentration under RCP 8.5 changed in a discontinuous trajectory with rather mild changes until the middle of the century, but intense and accelerating decrease afterwards (Fig. 4). After 2080, the MOM concentration, under RCP 8.5, was projected to decrease below 1 mg L^−1^ inducing severe metalimnetic hypoxia (Fig. 4).

**Table 2:** Projected trends of hydrodynamic and water quality variables in the twenty-first century, driven by three climate scenarios under the current withdrawal, and an additional scenario under RCP8.5 using surface withdrawal (SW)

	RCP2.6	RCP6.0	RCP8.5	RCP8.5 (SW)
Water Temp (°C decade^−1^)				
1 m depth	0.07 (*p* < 0.001)	0.29 (*p* < 0.001)	0.47 (*p* < 0.001)	0.45 (*p* < 0.001)
10 m depth	0.05 (*p* < 0.001)	0.16 (*p* < 0.001)	0.32 (*p* < 0.001)	0.27 (*p* < 0.001)
60 m depth	0.02 (*p* = 0.009)	0.06 (*p* < 0.001)	0.19 (*p* < 0.001)	0.05 (*p* = 0.009)
DO (mg L^−1^ decade^−1^)				
1 m depth	− 0.01 (*p* < 0.001)	− 0.07 (*p* < 0.001)	− 0.11 (*p* < 0.001)	− 0.10 (*p* < 0.001)
MOM	− 0.05 (*p* = 0.04)	− 0.27 (*p* < 0.001)	− 0.45 (*p* < 0.001)	− 0.23 (*p* = 0.04)
Below 35 m depth	− 0.02 (*p* < 0.001)	− 0.09 (*p* < 0.001)	− 0.22 (*p* < 0.001)	− 0.19 (*p* < 0.001)
Phosphate (mg L^−1^ decade^−1^)				
Vertical average	ns	2.8 × 10^–5^ (*p* < 0.001)	1.9 × 10^–4^ (*p* < 0.001)	3.6 × 10^–5^ (*p* < 0.001)
Diatoms (µg chl-a L^−1^ decade^−1^)				
Top 15 m depth	ns	ns	0.01 (*p* < 0.001)	ns
*P. rubescens* (µg chl-a L^−1^ decade^−1^)				
Top 15 m depth	ns	0.05 (*p* < 0.001)	0.16 (*p* < 0.001)	0.06 (*p* < 0.001)

In the table, *p* values are drawn from Mann–Kendall test, and the values in front indicate the changing slope from Theil–Sen method. Values represent averages over all four GCMs, and ns refers to not significant (*DO* dissolved oxygen, *MOM* metalimnetic oxygen minima, *RCP* representative concentration pathway)

Higher water temperatures and intensified oxygen depletion caused phosphate concentration in the reservoir to increase (Fig. S13) due to accelerating internal loading from mineralization and sediment release. Annually averaged phosphate concentration in the vertical direction increased by 2.8 × 10^–5^ mg L^−1^ decade^−1^ (RCP6.0) and 1.9 × 10^–4^ mg L^−1^ decade^−1^ (RCP8.5) but remained stable under the weak warming under RCP2.6 (Table 2). The maximum concentration in RCP8.5 reached 0.06 mg L^−1^ after 2090. By comparison, under RCP2.6, the annual average concentration was around 0.002 mg L^−1^ over the whole century (Fig. S13). Our results indicated that silicate and nitrate are less affected by climate change than phosphate (Figs. S14, S15). The seasonal distribution of their concentrations, under all three RCPs, remained rather constant during the whole century, with much lower values at the surface than at the bottom. Despite the pattern above, we found that driven by RCP8.5, in Period 85–99, a weak (but nevertheless clear) increase occurred at the end of stratification for the hypolimnetic silicate concentration, with the value reaching 4 mg L^−1^. In contrast, the corresponding nitrate concentration decreased to 0.6 mg L^−1^ (Fig. S14).

**Fig. 5:**
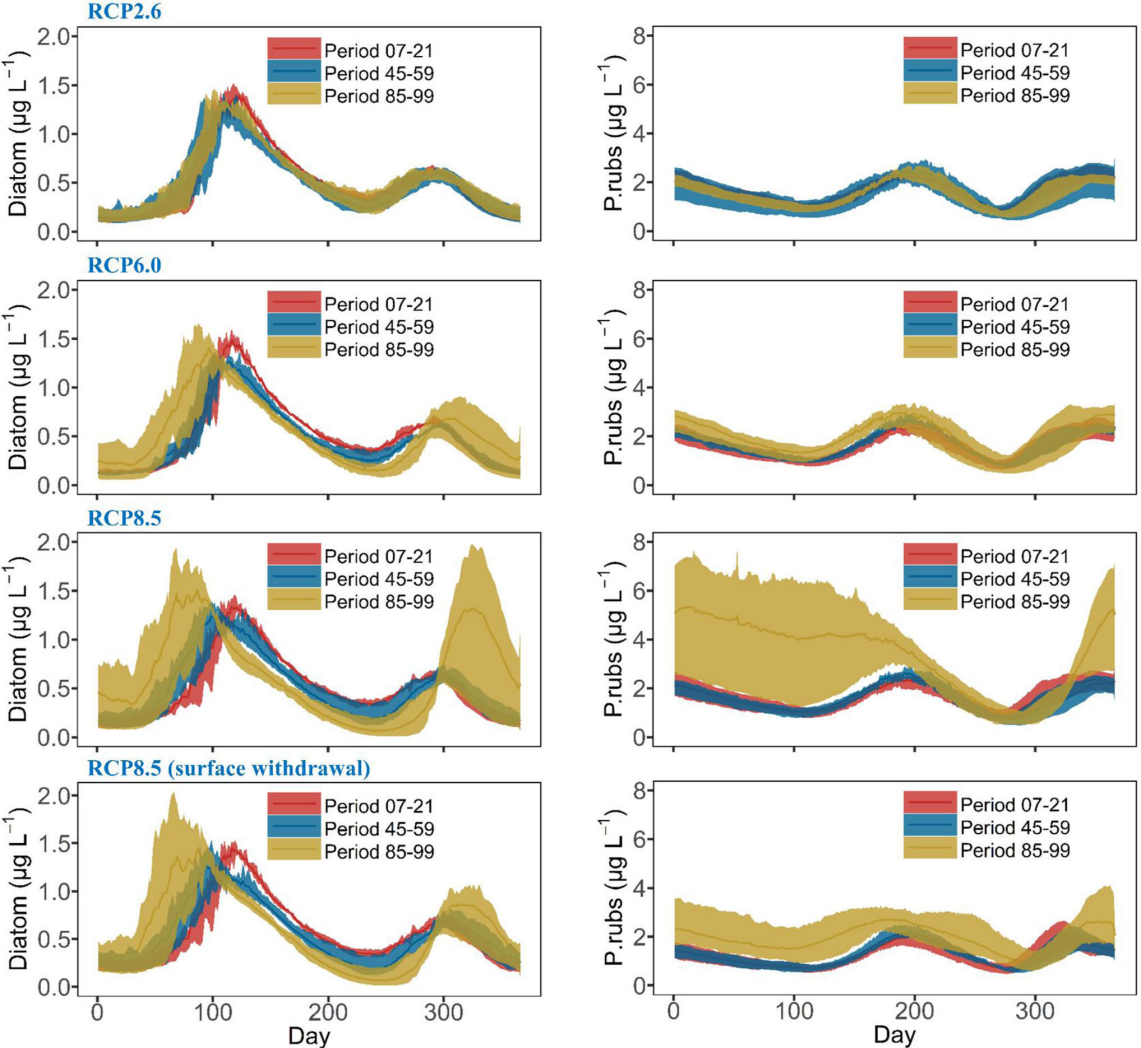
Future projections of diatoms (left) and *P. rubescens* (right) concentration in the top 15 m, for Rappbode Reservoir, during 3 periods (Period 07–21: from 2007 to 2021, Period 45–59: from 2045 to 2059, Period 85–99: from 2085 to 2099) under RCP2.6 (1st line), RCP6.0 (2nd line), RCP8.5 (3rd line) and RCP8.5 driven by the SW (4th line). Lines indicate the ensemble average results, for every Julian day, driven by four climate models, the shaded areas indicate the minimum and maximum results of the four means over 15 years of the four ensemble members

Comparable results were projected for phytoplankton concentrations, with the strongest changes under RCP 8.5, where annual average concentration in the top 15 m (the growth layer for the phytoplankton (Mi et al. 2020a)) increased at a rate of 0.16 µg L^−1^ decade^−1^ and 0.01 µg L^−1^ decade^−1^ for *P. rubescens* and diatoms, respectively (Table 2, Fig. S16). At weaker warming under RCP6.0, *P. rubescens* still increased by a rate of 0.05 µg L^−1^ decade^−1^ and no significant changes occurred for diatoms (Table 2). Concentration for both phytoplankton groups kept rather steady for the whole century under RCP2.6 (Table 2). Moreover, under RCP2.6 and 6.0, the concurrent succession patterns were conserved with a stable spring diatom bloom and a metalimnetic summer bloom of *P. rubescens*, while the winter was projected to be relatively dormant (Fig. 5). A shifting succession is projected for RCP 8.5 towards the end of twenty-first century, when the reservoir would also harbour overwintering phytoplankton populations, with *P. rubescens* dominating over diatoms (Fig. 5).

### Effects of alternative withdrawal strategies under future climate warming

Withdrawing the downstream discharge from deep layers intensifies warming (Table 2), whereby two processes come into play: (i) removing of the coldest layers and (ii) fast deepening of the epilimnion leading to maximum heat uptake. SW, instead, protects the cold hypolimnion from being withdrawn and keeps the epilimnion thinner, which lead to lower average heat contents and less pronounced deep layers warming (Fig. S17). Under RCP8.5, the water was projected to warm by 0.47 °C decade^−1^ at 1 m depth (annual ensemble means), 0.32 °C decade^−1^ at 10 m depth and 0.19 °C decade^−1^ at 60 m depth under the current withdrawal. By comparison, the rate decreased to 0.45 °C decade^−1^ (1 m), 0.27 °C decade^−1^ (10 m) and only 0.05 °C decade^−1^ (60 m) under the SW, indicating that SW increases the resistance of deep waters to future climate warming.

Moreover, the SW strategy significantly mitigated metalimnetic oxygen depletion under RCP8.5 (Fig. S18). The decreasing rate of MOM concentration under SW is nearly half of that under the current withdrawal (Table 2). As a consequence, DO concentration in the MOM remained above 2 mg L^−1^ under SW and severe metalimnetic hypoxia (< 1 mg L^−1^) was largely avoided during the century. In contrast, hypolimnetic DO concentration under the two strategies was similar over the whole century, albeit with slightly lower decreasing rates under the SW.

**Fig. 6:**
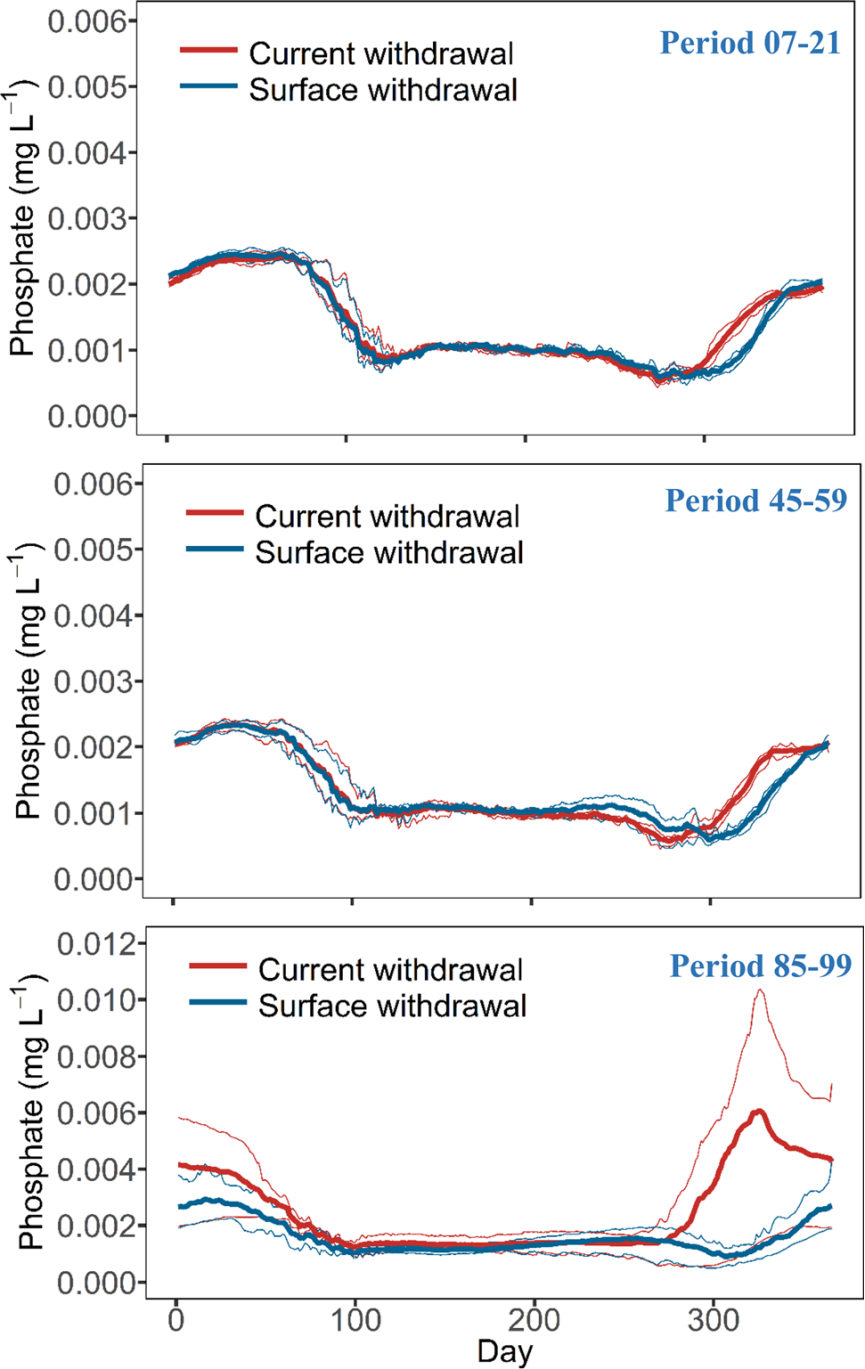
Future projections of phosphate concentration in the top 15 m, for Rappbode Reservoir, during 3 periods (Period 07–21: from 2007 to 2021, Period 45–59: from 2045 to 2059, Period 85–99: from 2085 to 2099) driven by the current and SW strategy under RCP8.5. The thick lines indicate the annual ensemble average results driven by four climate models and the thin lines indicate the annual minimum and maximum results of the four means over 15 years of the four ensemble members

Surface withdrawal also significantly suppressed the increase of phosphate concentration under future climate warming (RCP8.5, see Fig. S19 and Table 2). This is best reflected by winter phosphate concentration at the surface, which reached 0.006 mg L^−1^ at the end of the century (Period 85–99) under the current withdrawal, but decreased to 0.003 mg L^−1^ under the SW (Fig. 6).

The effects of SW on temperature, oxygen and phosphate also cascade down to phytoplankton as this strategy suppressed the winter blooms under climate warming (Fig. 5 and Table S3). In particular, the highest winter concentrations in Period 85–99 under the SW were 0.7 µg L^−1^ (diatoms) and 2.3 µg L^−1^ (*P. rubescens*), which are much lower than those under the current withdrawal strategy (1.8 µg L^−1^ and 5.8 µg L^−1^, respectively). It is also interesting to see that the intra-ensemble variability of phytoplankton projections shrank under the SW, leading to lower uncertainties associated with the different climate forcing.

## Discussion

### Cascading effects of future climate projection on reservoir ecosystems

Our results indicate increasing warming impacts on physical, chemical and biological variables from RCP2.6 to RCP8.5, and point to a strong need to slow the climate warming rate as far as possible to protect ecosystem health and water security. An important finding in this respect is that under RCP8.5, not only the surface but also the bottom temperatures were projected to increase significantly, with the latter reaching 7 °C at the end of the century (Fig. 2). This was unexpected since it has been suggested that deep temperatures in dimictic lakes should show little warming under future climate projections (Tan et al. 2018). Our results suggest that the deep-water warming could be due to the combined effect of climate warming and reservoir management. During normal operation, all water is abstracted from the hypolimnion, allowing the thermocline to deepen during the stratification period and the reservoir to take up more heat (Carr et al. 2019). Generally, the thermocline acts as a barrier to deep-water heating in reservoirs but if warming and hypolimnetic water withdrawal act together, heat penetration to deeper water is faster and more intense (Weber et al. 2017). For this reason, a changing management can help mitigate climate change impacts on the reservoir. Besides this adaptive withdrawal management, our results also emphasize that decelerating climate warming is the best strategy for aquatic ecosystem protection as only the worst-case scenario of RCP 8.5 showed severe negative impacts, while all other less intense warming scenarios showed far lower impacts. In fact, going from RCP 6.0 to RCP8.5 is really changing system characteristics (e.g. bottom temperature) and may represent a turning point.

Inverse stratification in winter, which defined as the number of days with surface temperature being 1 °C less than bottom temperature (Mi et al. 2019), strongly decreased under RCP8.5 and will nearly disappear after 2050 (Fig. S20). The vanishing of inverse stratification signals a transition from dimictic to a warm monomictic thermal regime. In the dimictic state, heat accumulated during each summer is completely lost and temperature gradually returns to around 4 °C at the mixing period (Boehrer and Schultze 2008). Warm monomictic lakes on the other hand can mix at temperatures above 4 °C, and under climate warming, extra heat accumulated in summer can be carried over into the following year (Lewis Jr. et al. 2019). As for Rappbode Reservoir driven by the current withdrawal strategy, the mixing temperature at the end of twenty-first century would be as high as 7 °C under RCP8.5 (Fig. S20) which significantly fuels the heat storage beneath water surface and enhances deep warming.

**Fig. 7:**
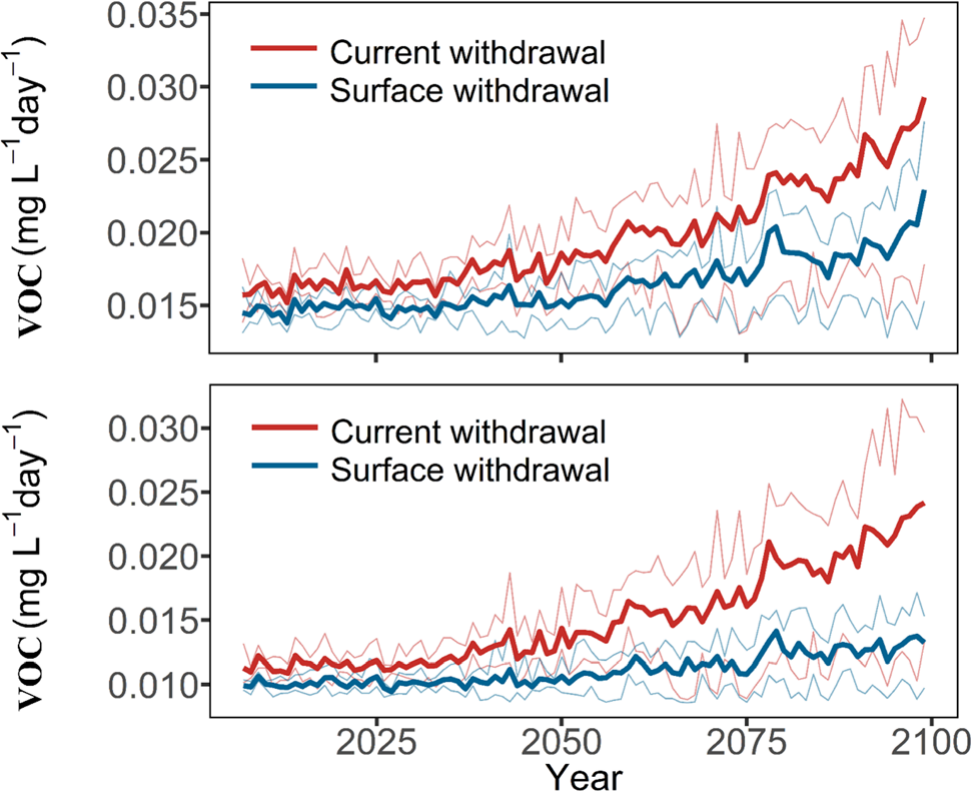
Future projections of the volumetric oxygen consumption (VOC, including sediment- and water-borne oxygen consumption) rate, for Rappbode Reservoir, in the upper (top) and lower (below) 35 m driven by the current and SW strategy under RCP8.5. The thick lines indicate the annual ensemble average results driven by four climate models and the thin lines indicate the annual minimum and maximum results from four means of the ensemble members

The future development of DO concentration is connected with the development of water temperature, with strongly adverse effects under RCP8.5 and far weaker changes under RCP2.6. We further extracted oxygen consumption rates from nitrification, organic matter decay and sediment oxygen demand (SOD) to illustrate which factors drive the oxygen decline. Our results indicated that the three rates were rather stable in the first half of the twenty-first century, but they strongly increased at the end of the century. In Periods 07–21 and 45–59 under RCP8.5, DO consumption from organic matter (OM) decomposition, for example, was always below 0.03 mg L^−1^ day^−1^ during the stratified period, and approaching 0 from day 300 onwards (Fig. S21). During Period 85–99, by comparison, the maximum rate could reach 0.04 mg L^−1^ day^−1^ during the stratified period, and 0.01 mg L^−1^ day^−1^ in winter. The effect of DO consumption from nitrification was similar (see Fig. S22), with the maximum peaking at 0.02 mg L^−1^ day^−1^ by the end of twenty-first century. The results also showed that SOD contributed more to DO consumption than the other two processes, reaching 0.08 mg L^−1^ day^−1^ at the end of stratification during the Period 85–99 (Fig. S23). Given all that, this enhancement of volumetric oxygen consumption (i.e. VOC, see “Adaptive management by alternative withdrawal strategies under future climate warming” section) rate under RCP8.5 is driving the strong decreases of oxygen concentration in the water column (Fig. 7). The low oxygen conditions then in turn induce the next step in the eutrophication cascade by phosphorus release. In parallel, silicate release and denitrification are intensifying as reflected by the changes of silicate and nitrate under RCP8.5 (see “Response of aquatic ecosystems to projected climate change” section). Such developments highly interfere with the drinking water supply, e.g. through the release of manganese or iron (Müller et al. 2012), and can be effectively prevented if warming in the twenty-first century is limited (e.g. RCP2.6).

Previous work always focussed on long-term changes of oxygen in the hypolimnion under different climate conditions (e.g. Zhang et al. 2015; Schwefel et al. 2016), whereas oxygen in the metalimnion has been largely overlooked. Our study shows that metalimnetic DO is highly sensitive to climate change, and there are good reasons to assume loss of oxygen in the metalimnion can also have profound effects on aquatic ecosystems. Firstly although with a relatively low ratio of sediment area to water volume, the higher temperature in the metalimnion than in the hypolimnion would still facilitate substantial nutrients release from the sediment following the occurrence of metalimnetic hypoxia (Yang et al. 2023). The nutrients are more easily accessible by algae in the productive metalimnetic zone than those from the hypolimnion (Falkowski and Raven 2013). Indeed our results indicate that in Rappbode Reservoir, lower metalimnetic DO concentrations under RCP8.5 coincide with rising phosphate concentration, which opens the niche for *P. rubescens* and supports eutrophication also in winter (Fig. 5). Under RCP8.5, winter will not stand out as a period with the minimum algae biomass, a pattern which is quite close to deep waters in the Mediterranean zone (Moustaka‐Gouni et al. 2014) rather than the temperate zone. Secondly, low DO in the metalimnion has a severe impact on biota, particularly fish, as it reduces their suitable habitat and the overlap between the temperature and oxygen niches (Coutant 1985) potentially leading to fish kills.

**Fig. 8:**
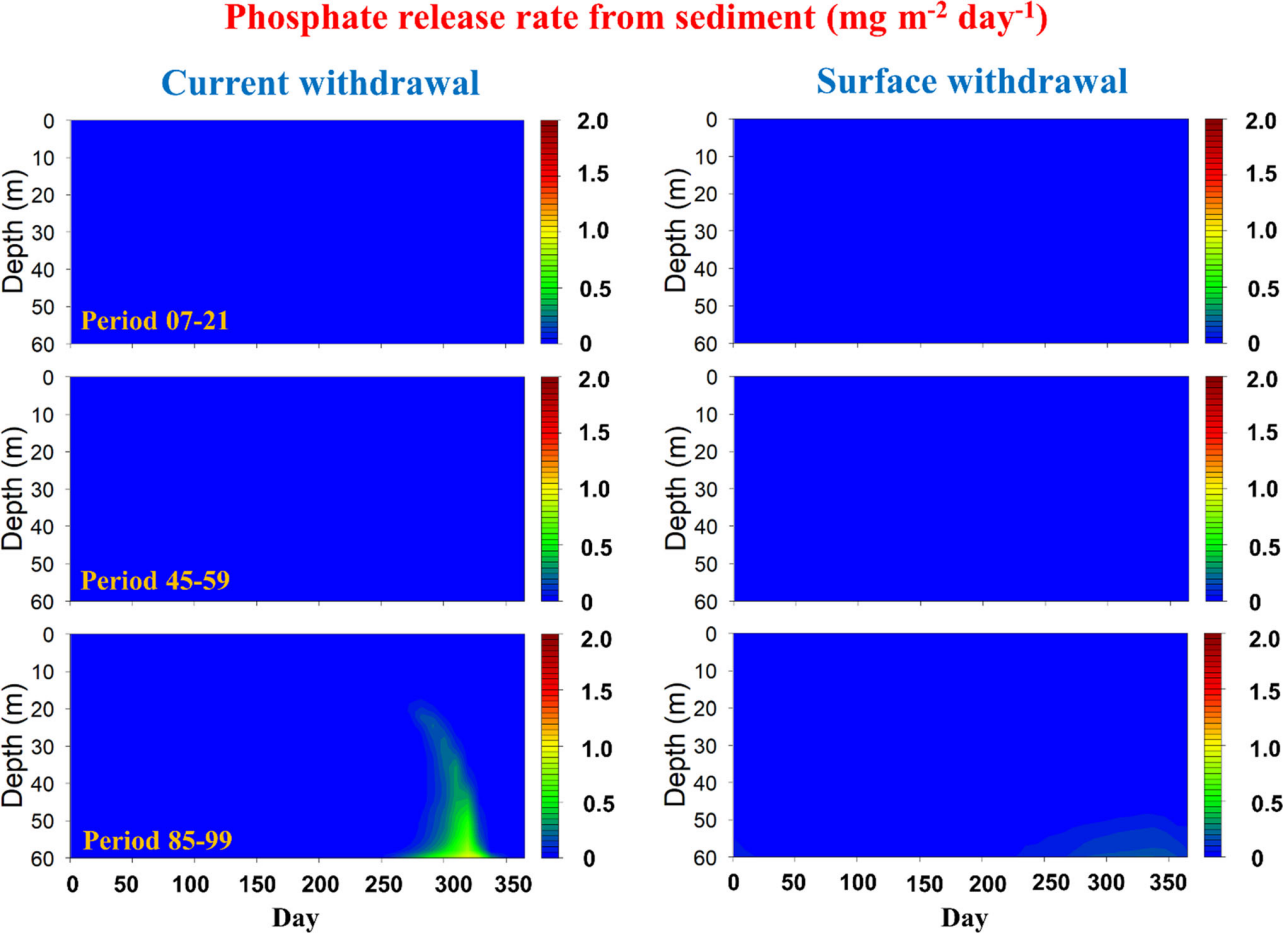
Phosphate release rate from sediment driven by the current (left) and surface (right) withdrawal strategy under RCP8.5 (seasonal ensemble averages over 15 simulated years as indicated in the left panels)

To confirm our findings, we then extracted the phosphate fluxes by different processes from the simulation output. Clearly driven by the coupled effects of high water temperature and low DO conditions under RCP8.5, phosphate release from the sediment significantly increased during Period 85–99 compared to the time before (Fig. 8). At the end of stratification, it reached 1.0 mg m^−2^ day^−1^ in the hypolimnion, and this high value expanded into the upper layer which can reach 0.5 mg m^−2^ day^−1^ even at 20 m depth (Fig. 8). During Period 85–99, our results also suggested an increasing phosphate production rate from OM decay in winter (Fig. S24), which should be due to the OM decomposition derived from dead algae.

One of the key messages from our study is that blooms of *P. rubescens* (cyanobacteria) will intensify under strong warming, with a high biomass even occurring in winter (Fig. 5). This fits well with empirical observations in other lakes, for example, Lac du Bourget (Jacquet et al. 2005) and Lake Geneva (Gallina et al. 2017). Applying different statistical methods to the long-term monitoring data in Lake Stechlin, which is around 200 km away from our study site, a recent study by Kröger et al. (2023) illustrated surface water warming, a prolonged stratification period and internal P release are the critical mechanisms driving the increase in *P. rubescens* blooms. Their conclusion is quite comparable to what we found using water quality simulations. According to the simulation results, we also found a strong prolongation of stratification duration under RCP8.5 (3.8 days decade^−1^), which should be considered as another driver facilitating *P. rubescens* growth in Rappbode Reservoir.

We finally want to stress that *P. rubescens* is able to regulate its buoyancy (Walsby et al. 2004). This physiological property is only indirectly included in the model, although Kerimoglu et al. (2017) and Fenocchi et al. (2019) applied similar approaches. If the buoyancy control is explicitly included in the model, we expect a further increase of *P. rubescens* under RCP8.5 since its buoyancy cells float better in more stagnant water with increasing stratification duration and water column stability (Carey et al. 2012).

**Fig. 9:**
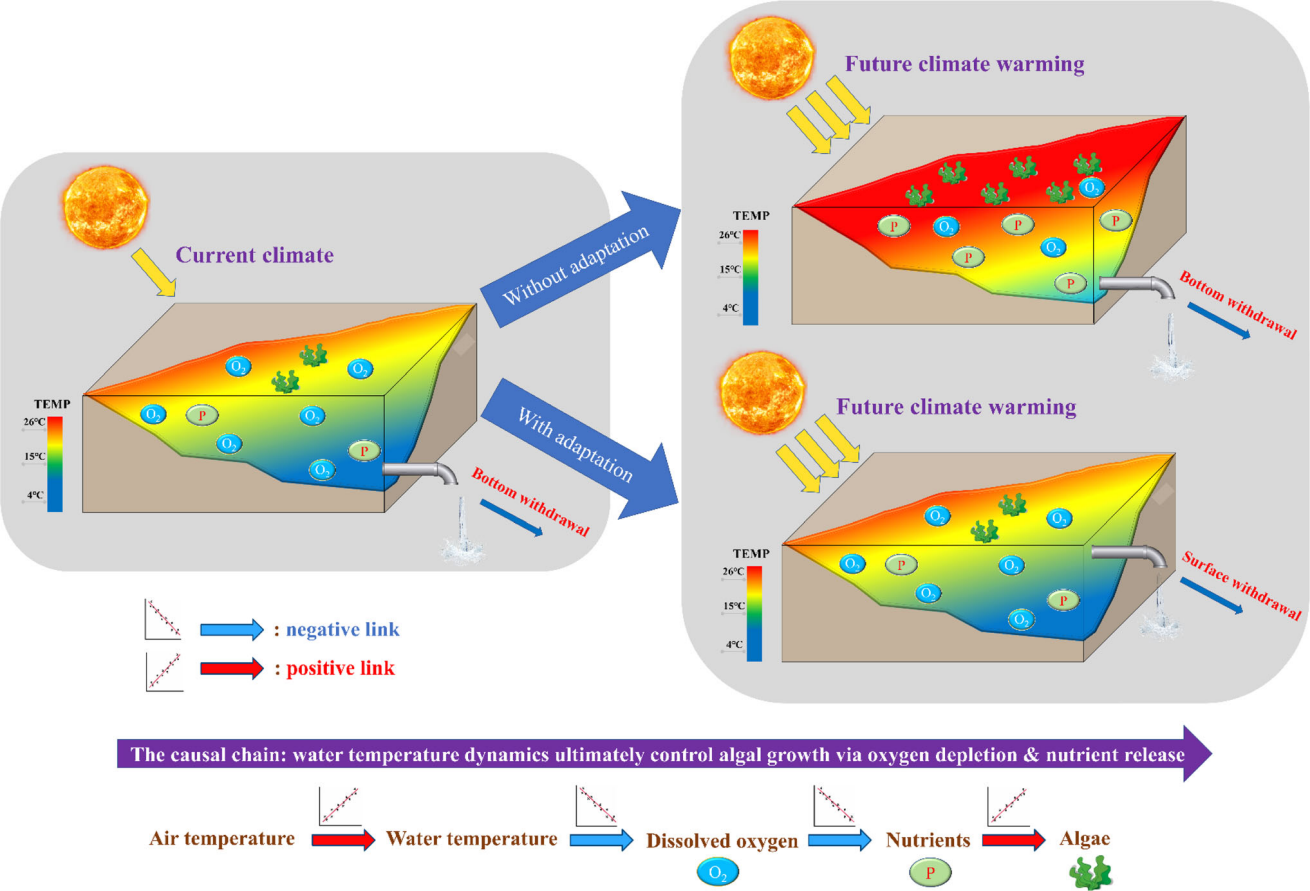
Conceptual framework visualizing the “cascading climate effect” on reservoir ecosystems, under the surface and bottom withdrawal strategy. The causal chain shown at the bottom summarizes the mode of action from physical variables towards biogeochemical and ecological water quality variables

In conclusion, our results illustrated the “cascading climate effect” propagating through the aquatic ecosystem, triggered by rising temperatures, accelerating mineralization and nutrient release and finally supporting eutrophication and cyanobacterial bloom (see Fig. 9). The cascading effect has been documented in empirical studies (e.g. Pettersson et al. 2010; Rigosi et al. 2014; Vystavna et al. 2017), and we further extended and quantified it under ensemble future climate projections. As a consequence, future climate warming and eutrophication are self-reinforcing, and synergistically deteriorate water quality. This cascade not only provides a process-based framework for elucidating climate change effects, but also gives guidance for stakeholders to deal with projected climate warming: optimizing management strategies to reduce warming of the water (the top-down approach) will effectively mitigate negative impacts of warming on aquatic ecosystems.

### Application of alternative withdrawal strategies to mitigate the effects of climate warming

Our results confirmed that SW can be an effective strategy to avoid the cascading climate-induced eutrophication effect by reducing the warming rate of the whole water column. Driven by the SW strategy, Rappbode Reservoir can always mix between 4 and 5 °C even at the end of the century (i.e. much lower than the temperature under the hypolimnetic withdrawal), which helps the water keep its dimictic state and effectively mitigates deep warming under RCP8.5 (Fig. S25). This cooling effect cascades through the ecosystem by suppressing DO consumption rates from OM decay (Fig. S21), nitrification (Fig. S22) and SOD (Fig. S23) since all of them are highly temperature-dependent. Besides, SW also helps weakening the release rate of phosphate from sediment and from OM decay and maintain its seasonal pattern, which barely changed throughout the whole century under RCP8.5 (Figs. 8, S24). Under this condition, the alternative withdrawal strategy indeed prevents the reservoir from occurring winter algal blooms and cyanobacterial dominance driven by intense climate warming, with a much lower *P. rubescens* biomass compared to that under the current withdrawal strategy (Fig. 5). The withdrawal management is therefore a prime instrument for reservoir managers to adapt reservoirs to climate changes, alleviate water quality deterioration and support water security (Fig. 9). Aside from that, our research also indicates coupled physical-ecological models can serve as valuable planning tools for enabling this knowledge-based reservoir operation.

Conclusion from our research agreed well with the inference from Lewis Jr. et al. (2019), indicating hypolimnetic withdrawal, which is currently used in most of reservoirs, may intensify the climate warming effects on water temperatures which can cause severe water quality problems (e.g. hypolimnetic anoxia, growth of harmful algae and so on). Such problems are faced by many temperate reservoirs (Woolway et al. 2020) and the adaptive potential of selective withdrawal strategies to mitigate climate warming effects is also relevant for other water bodies and their stakeholders. From this perspective, therefore, the importance of our research should be far beyond the individual case of Rappbode Reservoir. Besides, further potential of selective withdrawal is associated with exporting water of bad quality (e.g. algal rich) to downstream rivers in order to protect water quality of the drinking water resource (Mi et al. 2022).

Selective withdrawal can have complex effects on DO dynamics due to two counteracting effects of the strategy. Previous studies, on the one hand, reported the mitigation of hypoxia driven by bottom withdrawal (Weber et al. 2017; Aghasian et al. 2019), due to an advective downward flux of oxygen and shrinkage of the hypoxic layer. Some others, however, illustrated the opposite showing that SW can help achieve the goal in stratified waters like Lake Diefenbaker (Canada, Carr et al. 2019), Lake Kariba (Zambia and Zimbabwe, Calamita et al. 2021) and the El Carrizal Reservoir (Argentina, León et al. 2016), while by contrast the deep withdrawal can even worsen hypoxia by increasing hypolimnetic temperature and DO consumption rates. According to these opposing effects, we derived the idea of a hybrid withdrawal mode, i.e. using SW during early and mid stratification, and bottom withdrawal at the end of stratification, as the optimal method of suppressing the occurrence of hypolimnetic hypoxia. The rationale behind the hybrid withdrawal is that it can effectively decrease bottom water temperatures and meanwhile increase the duration of the mixing period to facilitate the vertical oxygen transfer, as has been verified by our recent study (see Mi et al. 2023). We assume it will be greatly valuable for the future studies to couple this hybrid mode with projected climate warming scenarios (e.g. RCP8.5) to provide a more solid basis for stakeholders to effectively reduce the risk of hypoxia in the future.

Besides the two options above (i.e. either surface or bottom withdrawal), also withdrawal from intermediate depths, i.e. out of the metalimnion or upper interior of the hypolimnion, can be a valuable topic to explore considering increased stratification intensity under future climate warming. Here, the buoyancy frequency (*N*^*2*^) during summer (i.e. from July to September) significantly increased under RCP8.5 driven by both classical bottom (1.8 × 10^–4^ s^−2^ decade^−1^, the annual ensemble mean) and surface (2.6 × 10^–4^ s^−2^ decade^−1^, see Fig. S26) withdrawal indicating a more stable stratification structure in future. We therefore expect that vertical heterogeneity in reservoirs will increase over time and gradients of water quality variables will become steeper and more variable, and this trend will make a more flexible, depth-variable water withdrawal even more interesting for the adaptive reservoir management.

### Limitation and outlook

The effect of climate change on water quality of lakes and reservoirs is harming water security and model tools like ours are required for making projections and identifying management measures. Our approach nevertheless suffers from uncertainties in climate projections and therefore follows an ensemble approach. We also note that for the high warming scenarios of RCP8.5, the physical and biogeochemical conditions in the reservoir changed beyond the conditions for which the model was calibrated/validated, and therefore, there is a significant risk that the ecosystem will react differently to what was projected from this study. Another critical issue is the transferability of model formulations. This refers not only to the physical domain, where maybe 3D models (e.g. EFDC, Delft3D or AEM3D) are required for complex lake morphologies, but also for the ecological model as many parameters differ between lakes and we still lack a “general lake ecosystem model” that works everywhere well.

Moreover, previous work based on in situ measurements has confirmed the importance of nutrient loading (and land-use changes) in shaping trophic and energetic dynamics for downstream waters (see Paul et al. 2021; Shi et al. 2020), while how the situation will be changed under future climate projections is still far less understood. In Rappbode Reservoir, the inflow is strongly regulated by three constructed pre-reservoirs, making it inappropriate for the related studies. Alternatively, we suppose it will be a quite interesting study to elucidate the combined effects of projected climate change and land-use change scenarios on lentic waters, with the inflow from natural origin. It is believed that by upscaling the research from a local to a regional perspective, results from such studies will comprehensively improve our understanding about the impacts of complex future conditions.

## Conclusions

- Intense climate warming (RCP8.5) will trigger a transition of thermal regime in Rappbode Reservoir from a dimictic to warm monomictic type, which fuels the heat storage beneath water surface and enhances deep warming. These effects, by comparison, become much weaker under the other two climate scenarios (RCP2.6 and 6.0).
- Our results illustrated cascading effects, driven by ensemble future climate projections, propagating through the aquatic ecosystem triggered by rising water temperatures, leading to accelerating mineralization and nutrient release, and finally supporting eutrophication and cyanobacterial blooms. This extends the conclusions from previous studies based on *in-situ* measurements.
- Under RCP8.5, winter will not stand out as a period with the minimum algae biomass in Rappbode Reservoir, a pattern which is quite close to deep waters in the Mediterranean zone rather than the temperate zone.
- By suppressing the increase of water temperatures, surface water withdrawal can be used as an effective way to weaken the increase of phosphate concentration, the occurrence of severe hypoxia, and winter cyanobacteria blooms under climate warming. This should provide scientific guidance for stakeholders to further optimize their management strategies in confronting potential climate change.

## Supplementary Information

Below is the link to the electronic supplementary material.

Supplementary file1 (PDF 19084 KB)
