# Supplementary material for: Cascading climate effects in deep reservoirs: Full assessment of physical and biogeochemical dynamics under ensemble climate projections and ways towards adaptation
Source: Ambio. 2023 Nov 8;54(3):385–401. doi: 10.1007/s13280-023-01950-0 (PMC11780243; doi:10.1007/s13280-023-01950-0)
Supplement: Supplementary file 1 — Supplementary file1 (PDF 19084 KB) [file 13280_2023_1950_MOESM1_ESM.pdf]

*Ambio*

Electronic Supplementary Material

Title**: Cascading climate effects in deep reservoirs: Full assessment of physical and biogeochemical dynamics under ensemble climate projections and ways towards adaptation**

Authors: Chenxi Mi, Tom Shatwell, Xiangzhen Kong, Karsten Rinke

## Phytoplankton groups defined in the model

The two phytoplankton groups (i.e. diatoms and *P. rubescens*) in the model are designed according to our long-term in-situ measurements by a multi-channel fluorescence probe (FluoroProbe, bbe moldaenke GmbH, Germany, serial number: 2101, please see Wentzky et al. (2019)), which indicate that these two functional groups cover most of the autotrophic phytoplankton biomass. This is supported also by long-term phytoplankton counts, which confirm that the diatom *Asterionella formosa* dominates the biomass during spring. Also other diatom species co-occur during this time, e.g. species of the genera *Tabellaria, Fragillaria, Cyclotella, Stephanodiscus* or *Synedra*. *P. rubescens* subsequently grows as the dominant species from late spring until autumn, at around a depth of 10 m every year. Although there are also mixotrophic algae abundant during specific times, their autotrophic contribution is expected to be rather small so that we did not include them as state variables in the model.

## Model setup for future inflow temperature and nutrient concentration

We built a piecewise linear regression model using the R package "segmented" (v1.6-4, Muggeo 2017) to describe the relationship between observed air temperature and measured inflow water temperature for 2014-2019. We then applied this model to project future inflow temperature under each climate scenario (following the method by Tan et al. (2018)) using projected air temperature. Inflow nutrient concentration measured in 2016, were used as inputs for each projected year, since its dynamics in 2016 appeared to be rather normal. This simplification is reasonable because inter-annual variability in inflow nutrients only marginally affects ecosystem dynamics in Rappbode Reservoir (Mi et al. 2020).

## R packages used for pre- and post-processing under RCP scenarios

We used the function “GAM” in the package “mgcv” (v1.9-0, Wood 2011) to establish the generalized additive model, which was used to calculate daily average inflow and outflow discharge (see section 2.4 in the main document). A Mann-Kendall test was used to check the temporal trend in time series data (via function “MannKendall” in the package “Kendall” (v2.2.1, McLeod 2022)) and Theil-Sen method to quantify the slope of changing trends (via function “TheilSen” in the package “robslopes” (v1.1.3, Dillencourt et al. 1992)). Besides we used the function “ts.buoyancy.freq” in the package “rLakeAnalyzer” (v 1.11.4.1, Winslow et al. 2019) to quantify buoyancy frequency at the thermocline.

## Procedure on model calibration and parameterization

As a rule of thumb, for the calibration we firstly calibrated parameters for water temperature dynamics via the traditional “trial and error” method, and then for the ecological processes. According to the official manual of W2 and previous studies (Cole and Wells 2006; Kobler et al. 2018; Sadeghian et al. 2018), wind sheltering (WSC), shading (SHADE) and light extinction coefficients (EXH2O) are highly lake specific and require local adaptation for the temperature simulation, which should be considered for the calibration. Here EXH2O was set as 0.45 $m^{-1}$ according to our measurements of underwater light distribution (LICOR-193SA underwater quantum sensor), and the other two were calibrated by manual adaptation. During this process, all other internal hydrodynamics parameters were kept unchanged because they have a solid empirical basis (Chuo et al. 2019).

More parameters were related to the ecological simulation, and our philosophy was to determine *a priori* as many parameters as possible. Two types of phytoplankton (i.e. diatoms

and *P. rubescens*) were included in the model, based on the seasonal dynamics of dominant species in Rappbode Reservoir (see Section 2.1). Our measurements indicated spring diatoms exhibit r-selected traits with moderate light requirements, high maximum growth rates, broad temperature range for the growth and low nutrient affinity, whereas *P. rubescens* should be classified as k-selected organisms with low growth rate, high tolerance to low light and temperate temperature optima, and also high nutrient affinity helping it to be competitive in nutrient limited waters (Kerimoglu et al. 2017). By linking such trait properties with values from previous studies, we then determined *a priori* most of parameters related to the two phytoplankton (Table S1) except four key parameters controlling their light-dependent growth kinetics (i.e. ASAT#1, ASAT#2, AG#1 and AG#2, please see Table S2 for details), which were manually calibrated within the reasonable range (Table S2). Finally, sediment oxygen demand (SOD) was fixed according to the oxygen consumption rate in hypolimnion calculated from oxygen concentration dynamics by using the method from Livingstone and Imboden (1996), which quantified the specific contribution of pelagic and sediment-related processes to the hypolimnetic oxygen dynamics. For all other parameters we either applied the values recommended in the user manual of W2 or from related studies (Table S1), and readers are referred to our previous work (Mi et al. 2020) for further details of the water quality calibration.

## Uncertainty for future projections

As shown in this and previous studies (Shatwell et al. 2019; Tan et al. 2018), the projection of aquatic ecosystems is sensitive to changes in climate forcings. Any variability in the meteorological inputs hence propagates through the hydrodynamic and ecological model and

induces changing values. In our study, we used the output from an ensemble of climate models to reflect the uncertainty in future climate projections. Also in our study, variability of projections around their mean, for both hydrodynamic and ecological variables, gradually increased over time, especially under RCP 8.5. It has been shown that thermal dynamics in lentic waters is mainly controlled by wind speed, air temperature and shortwave radiation (Boehrer and Schultze 2008). Under RCP 8.5, although wind speed, for Rappbode Reservoir, was projected quite close to each other from all four climate models (Fig. S27), shortwave radiation and air temperature from GFDL were significantly lower than for the other three models resulting in a big difference of water temperature projections. Similar pattern was also found for the projections of oxygen concentration, phosphate and *P.rubescens* biomass which are highly controlled by the thermal structure. All such results, consequently, illustrate the importance to analyze future changes of temperate waters driven by an ensemble of climate models (Schlabing et al. 2014).

Besides, the uncertainty can also be due to the choice of lake models with different dimension (e.g. 2D or 3D), cell structure (e.g. rectangular or trapezoidal cells) and discretization methods (e.g. finite-volume or finite-difference method, see Mi et al. (2019)). From our knowledge, there are already some studies analyzing this part of uncertainty for water temperatures (Feldbauer et al. 2022; Martynov et al. 2010), while very few of them focused on aquatic ecosystems. To fill in this gap, consequently, it is recommended as the next step to couple the ensemble of climate and lake models. By doing so, we believe conclusion drawn from the biogeochemical projections (especially under the strong warming scenario of RCP8.5) should be more promising.

Table S1. Values of *a priori* determined parameters including references

| Parameter | Description | Value | Reference |
|---|---|---|---|
| AR#1 | Dark respiration rate for P-rub ($day^{-1}$) | 0.02 | Brito et al. (2018) |
| AR#2 | Dark respiration rate for diatoms ($day^{-1}$) | 0.05 | Kobler et al. (2018) |
| AE#1 | Excretion rate for P-rub ($day^{-1}$) | 0.01 | Kerimoglu et al. (2017) |
| AE#2 | Excretion rate for diatoms ($day^{-1}$) | 0.05 | Cole and Wells (2006) |
| AM#1 | Mortality rate for P-rub ($day^{-1}$) | 0.005 | Carraro et al. (2012) |
| AM#2 | Mortality rate for diatoms ($day^{-1}$) | 0.05 | Sadeghian et al. (2018) |
| AS#1 | Settling rate for P-rub ($day^{-1}$) | 0.001 | Cole and Wells (2006) |
| AS#2 | Settling rate for diatoms ($day^{-1}$) | 0.05 | Chuo et al. (2019) |
| AHSP#1 | Half-saturation coefficient for phosphorus limited growth for P-rub ($g\ m^{-3}$) | 0.002 | Carraro et al. (2012) |
| AHSN#1 | Half-saturation coefficient for nitrogen limited growth for P-rub ($g\ m^{-3}$) | 0.005 | Bowie et al. (1985) |
| AHSN#2 | Half-saturation coefficient for nitrogen limited growth for diatoms ($g\ m^{-3}$) | 0.1 | Cole and Wells (2006) |
| AHSSI#2 | Half-saturation coefficient for silica limited growth for diatom ($g\ m^{-3}$) | 0.1 | Bowie et al. (1985) |
| AT1#1 | Lower temperature for P-rub growth (ºC) | 5 | Park et al. (2014) |
| AT1#2 | Lower temperature for diatoms growth (ºC) | 0 | Kobler et al. (2018) |
| AT2#1 | Lower temperature for maximum P-rub growth (ºC) | 10 | Bowie et al. (1985) |
| AT2#2 | Lower temperature for maximum diatoms growth (ºC) | 11 | Kobler et al. (2018) |
| AT3#1 | Upper temperature for maximum P-rub growth (ºC) | 14 | Fenocchi et al. (2019) |
| AT3#2 | Upper temperature for maximum diatoms growth (ºC) | 15 | Kobler et al. (2018) |
| AT4#1 | Upper temperature for P-rub growth (ºC) | 18 | Bowie et al. (1985) |
| AT4#2 | Upper temperature for diatoms growth (ºC) | 30 | Kobler et al. (2018) |
| ACHLA#1 | Ratio between P-rub biomass and chlorophyll a in terms of mg dry weight /μg chl a | 0.18 | Wentzky et al. (2019) |

| Parameter | Description | Value | Reference |
|---|---|---|---|
| ACHLA#2 | Ratio between diatoms biomass and chlorophyll a in terms of mg dry weight/µg chl a | 0.12 | Smith et al. (2014) |
| O2AG#1 | Oxygen stoichiometry for P-rub primary production (mg $O_2$/mg organic matter) | 1.1 | Wentzky et al. (2019) |
| O2AG#2 | Oxygen stoichiometry for diatoms primary production (mg $O_2$/mg organic matter) | 1.4 | Chuo et al. (2019) |
| O2AR#1 | Oxygen stoichiometry for P-rub primary respiration (mg $O_2$/mg organic matter) | 1.1 | Cole and Wells (2006) |
| O2AR#2 | Oxygen stoichiometry for diatoms primary respiration (mg $O_2$/mg organic matter) | 1.4 | Deliman and Gerald (2002) |
| ORGP | Stoichiometric equivalent between organic matter and phosphorus | 0.005 | Cole and Wells (2006) |
| ORGN | Stoichiometric equivalent between organic matter and nitrogen | 0.08 | Cole and Wells (2006) |
| ORGC | Stoichiometric equivalent between organic matter and carbon | 0.45 | Cole and Wells (2006) |
| ORGSI | Stoichiometric equivalent between organic matter and silica | 0.18 | Cole and Wells (2006) |
| POMS | Particulate organic matter settling rate (m $day^{-1}$) | 0.5 | Chuo et al. (2019) |
| PO4R | Sediment release rate of phosphorus, fraction of SOD | 0.015 | Chuo et al. (2019) |
| NH4R | Sediment release rate of ammonium, fraction of SOD | 0.15 | Chuo et al. (2019) |
| NH4DK | Ammonium decay rate ($day^{-1}$) | 0.15 | Brito et al. (2018) |
| NO3DK | Nitrate decay rate ($day^{-1}$) | 0.05 | Sadeghian et al. (2018) |
| DSIR | Dissolved silica sediment release rate, fraction of SOD | 0.1 | Cole and Wells (2006) |
| SODT1 | Lower temperature for SOD (ºC) | 4 | Cole and Wells (2006) |
| SODT2 | Upper temperature for SOD (ºC) | 30 | Cole and Wells (2006) |

| Parameter | Description | Value | Reference |
|---|---|---|---|
| SODK1 | Fraction of SOD at lower temperature | 0.1 | Cole and Wells (2006) |
| SODK2 | Fraction of SOD at upper temperature | 0.99 | Cole and Wells (2006) |

Table S2. The applied minimum and maximum values as well as their intervals for the calibrated parameters

| Parameters | Description | Minimum | Maximum | Interval | Calibrated values |
|---|---|---|---|---|---|
| AG #1 | Growth rate for P-rub ($day^{-1}$) | 0.35 | 0.65 | 0.05 | 0.55 |
| AG #2 | Growth rate for diatoms ($day^{-1}$) | 0.8 | 2.5 | 0.1 | 1.5 |
| ASAT#1 | Light saturation intensity at maximum photosynthetic rate for P-rub ($W\ m^{-2}$) | 4 | 15 | 1 | 8 |
| ASAT#2 | Light saturation intensity at maximum photosynthetic rate for diatoms ($W\ m^{-2}$) | 20 | 50 | 5 | 35 |

Table S3. Projections in key hydrodynamic and water quality variables driven by three climate scenarios under the current withdrawal, and RCP8.5 under the surface withdrawal (SW). Values in the table represent averages over all four GCMs during the start (i.e. Period 07-21, shorten as P 07-21) and end (i.e. Period 85-99, shorten as P 85-99) of 21st century. Here winter is defined as December, January and February (DO: dissolved oxygen, MOM: metalimnetic oxygen minima, RCP: representative concentration pathway).

| | **RCP2.6** | | **RCP6.0** | | **RCP8.5** | | **RCP8.5 (SW)** | |
|---|---|---|---|---|---|---|---|---|
| | **P 07-21** | **P 85-99** | **P 07-21** | **P 85-99** | **P 07-21** | **P 85-99** | **P 07-21** | **P 85-99** |
| **Water Temp (°C)** | | | | | | | | |
| 1 m depth | 11.55 | 12.31 | 11.41 | 13.52 | 11.58 | 15.23 | 11.32 | 14.83 |
| 10 m depth | 9.98 | 10.35 | 9.98 | 11.09 | 10.06 | 12.46 | 9.90 | 11.81 |
| 60 m depth | 4.80 | 4.95 | 4.84 | 5.38 | 4.85 | 6.31 | 4.43 | 5.04 |
| **DO (mg $L^{-1}$)** | | | | | | | | |
| MOM | 4.56 | 4.29 | 4.41 | 3.43 | 4.38 | 1.47 | 4.64 | 2.69 |
| Below 35 m depth | 9.95 | 9.75 | 9.91 | 9.24 | 9.91 | 7.91 | 9.39 | 7.48 |
| **Phosphate (mg $L^{-1}$)** | | | | | | | | |
| Surface | $1.3\times 10^{-3}$ | $1.4\times 10^{-3}$ | $1.4\times 10^{-3}$ | $1.5\times 10^{-3}$ | $1.3\times 10^{-3}$ | $2.5\times 10^{-3}$ | $1.3\times 10^{-3}$ | $1.5\times 10^{-3}$ |
| **Winter Diatoms (µg chl-a $L^{-1}$ decade$^{-1}$)** | | | | | | | | |
| Surface | 0.17 | 0.19 | 0.20 | 0.31 | 0.19 | 0.62 | 0.25 | 0.50 |
| **Winter *P.rubescens* (µg chl-a $L^{-1}$ decade$^{-1}$)** | | | | | | | | |
| Surface | 1.86 | 1.82 | 2.02 | 2.46 | 1.96 | 4.72 | 1.33 | 2.11 |

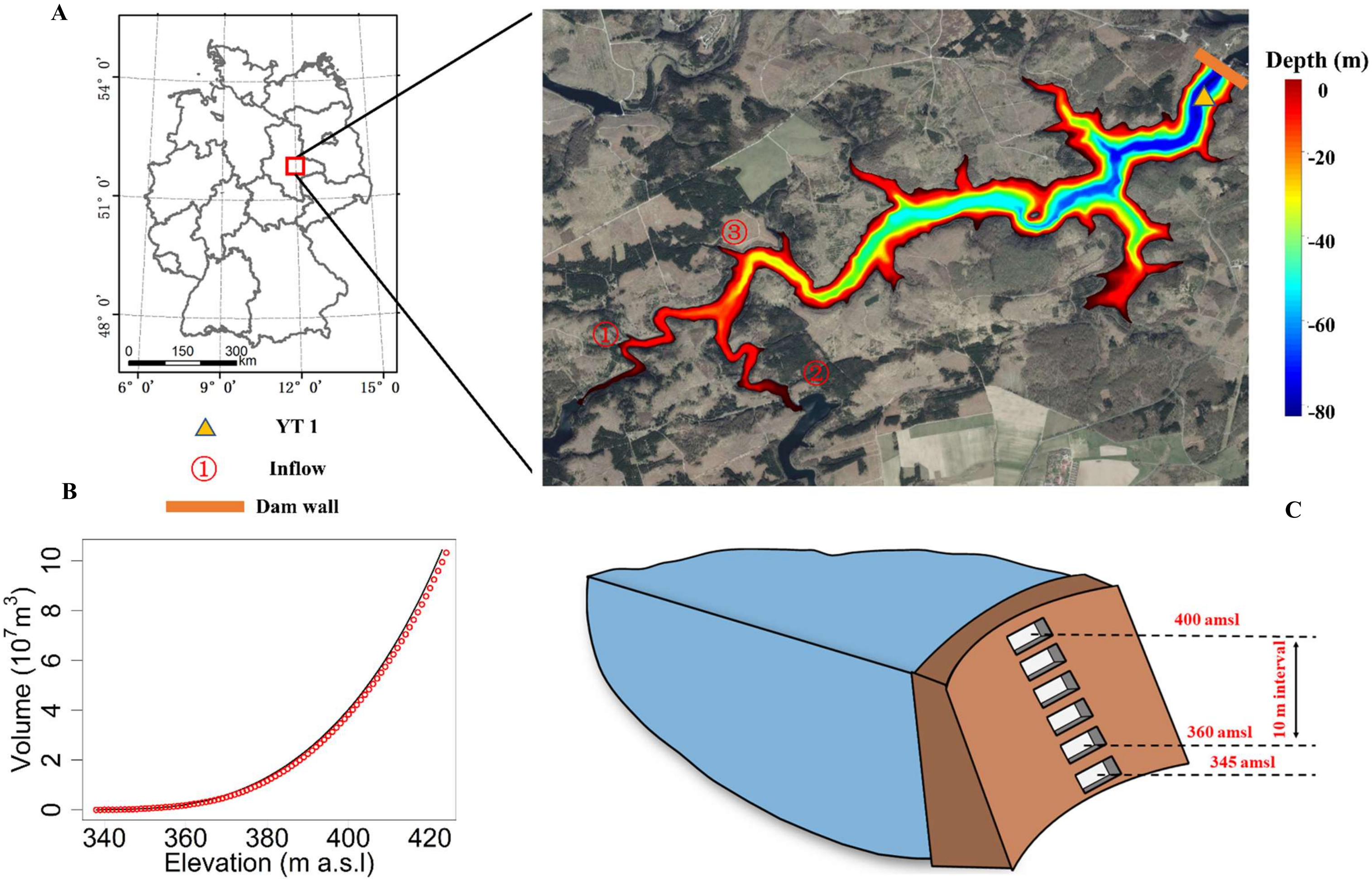


Fig. S1. (A) Map of Germany and the bathymetric of Rappbode Reservoir, with the point YT1 indicating the location of water quality measurements; (B) Relationship between elevation and volume for the reservoir, according to the measurements (red points) and established model (black line); (C) Layout of the selective withdrawal facility in the reservoir.

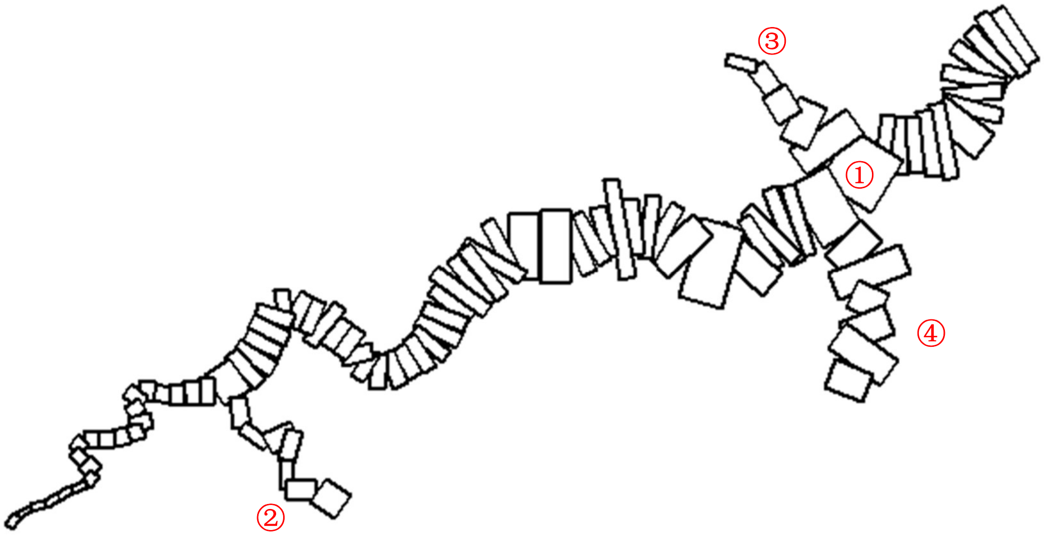


Fig. S2. Plan view of the grid structure for Rappbode Reservoir, with numbers showing the branch.

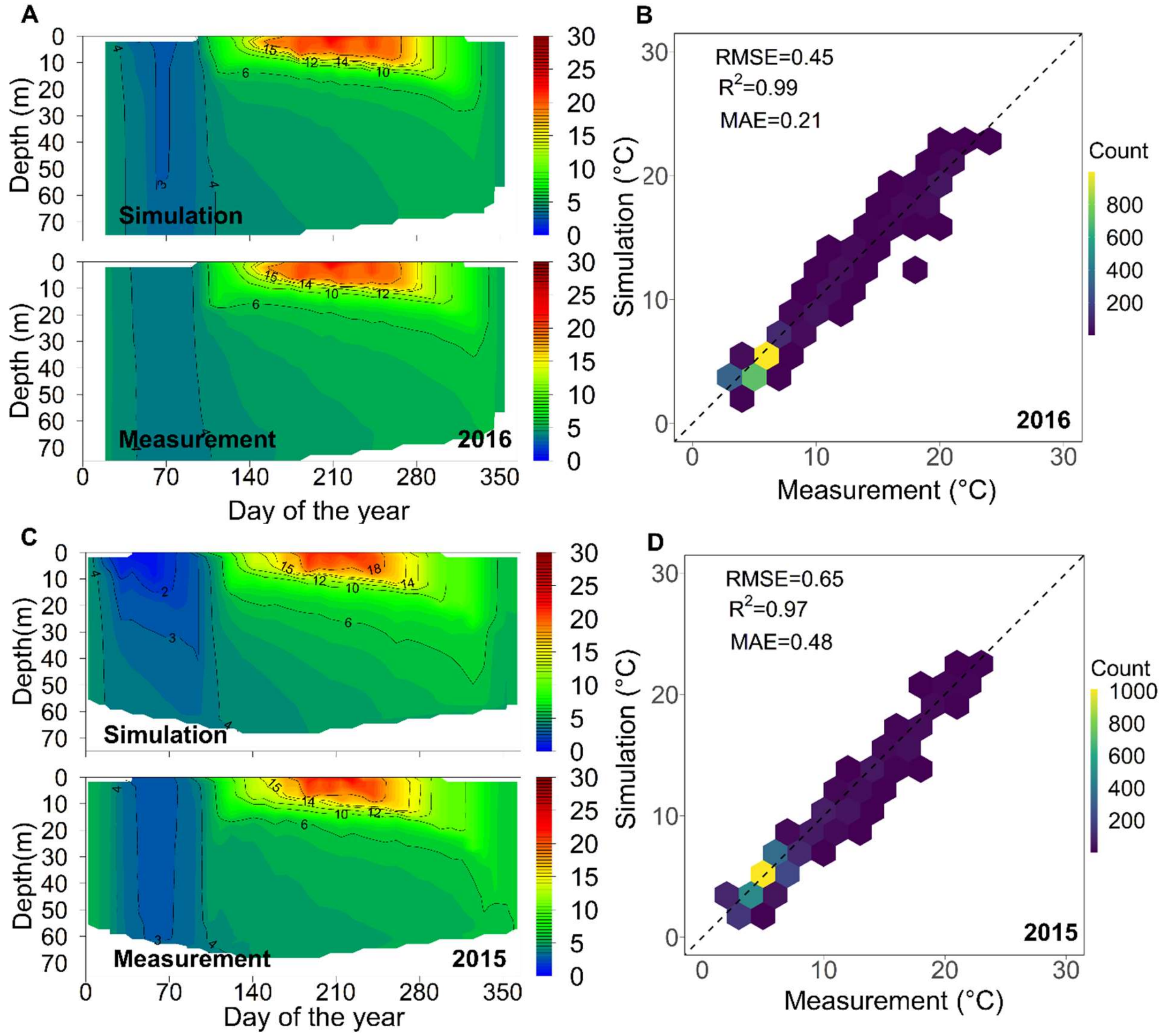


Fig. S3. Comparison between simulated and measured water temperature during the calibration (A and B) and validation (C and D) period. The color scale, in subfigure B and D, denotes the amount of samples per hexagon, and the straight line has a slope of one with an intercept of zero. The number at bottom-right shows the year of comparison.

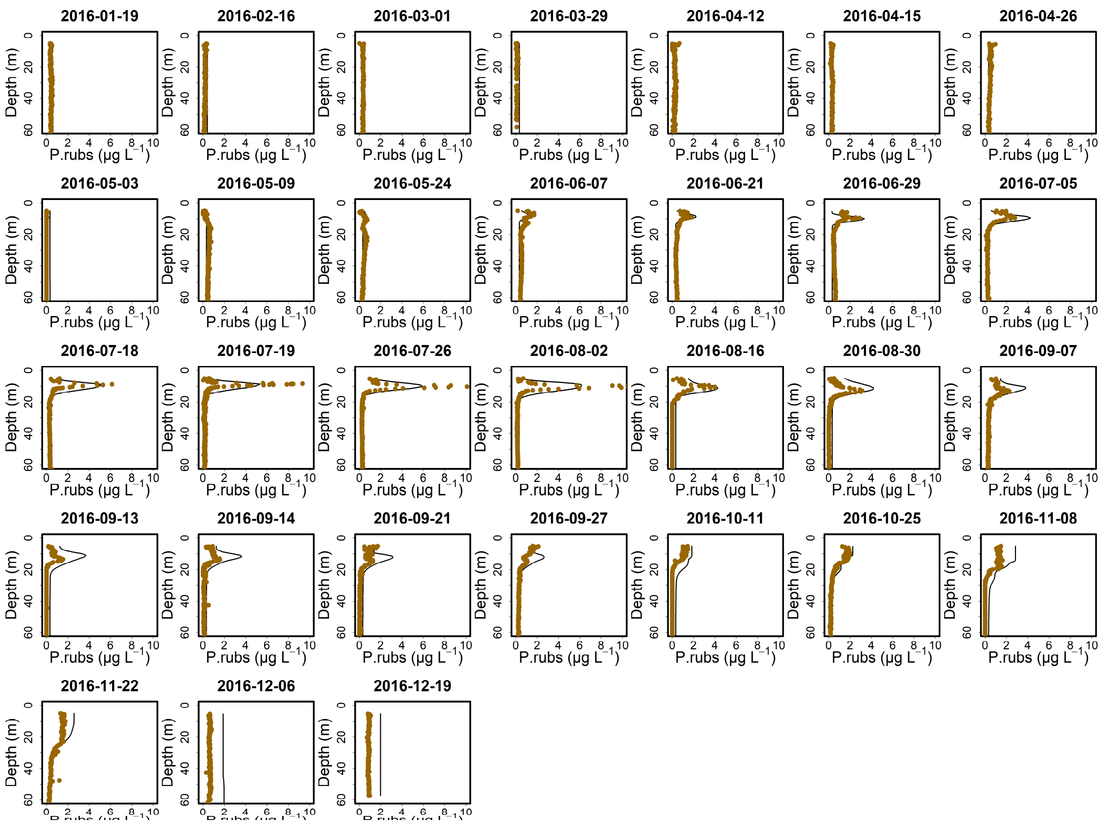


Fig. S4. Comparison between simulated (lines) and measured (points) *P. rubescens* during the calibration period.

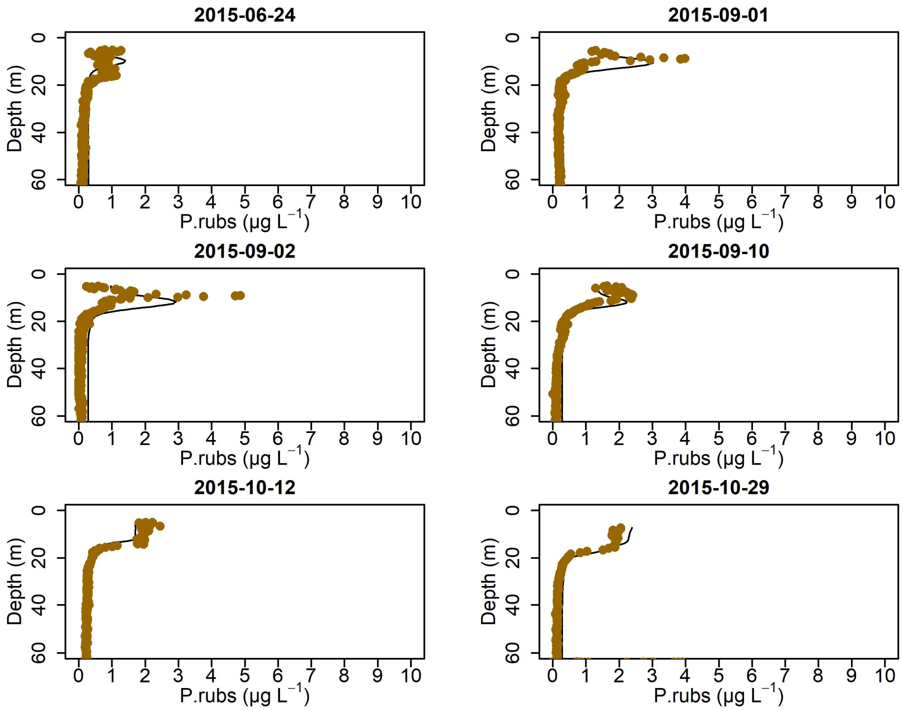


Fig. S5. The same as Fig. S4 but for the validation period.

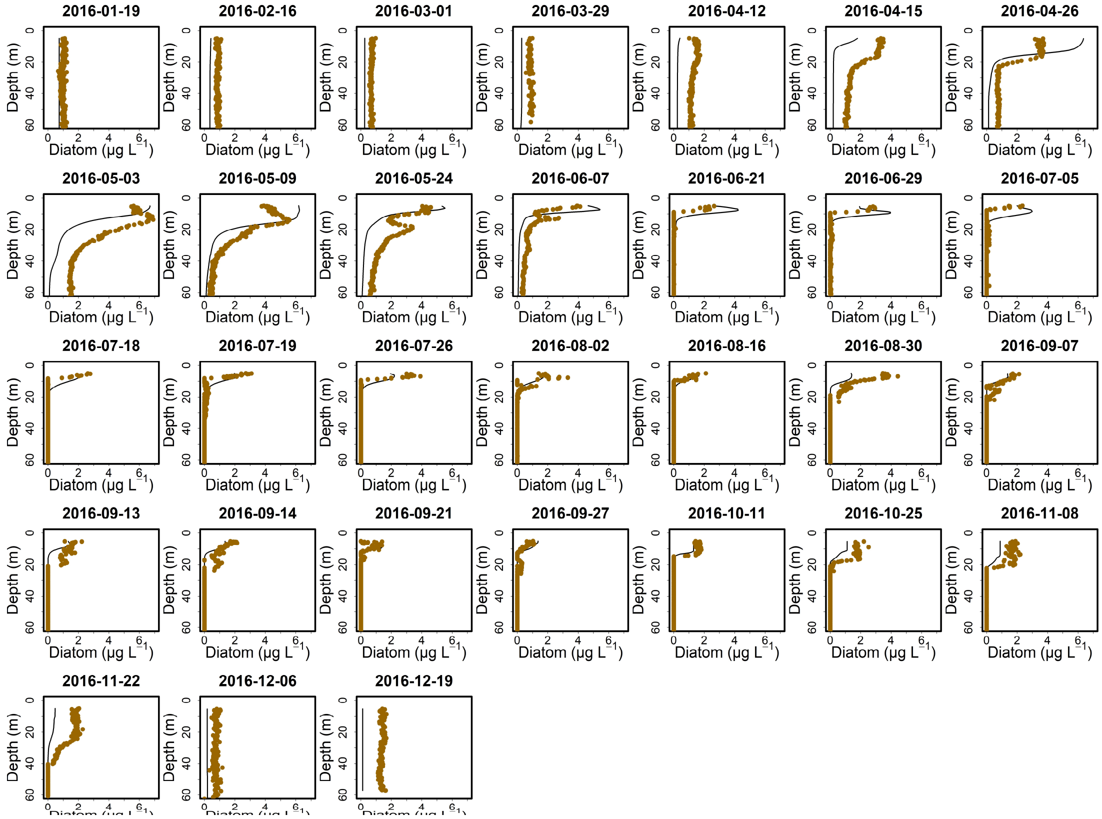


Fig. S6. Comparison between simulated (lines) and measured (points) diatoms during the calibration period.

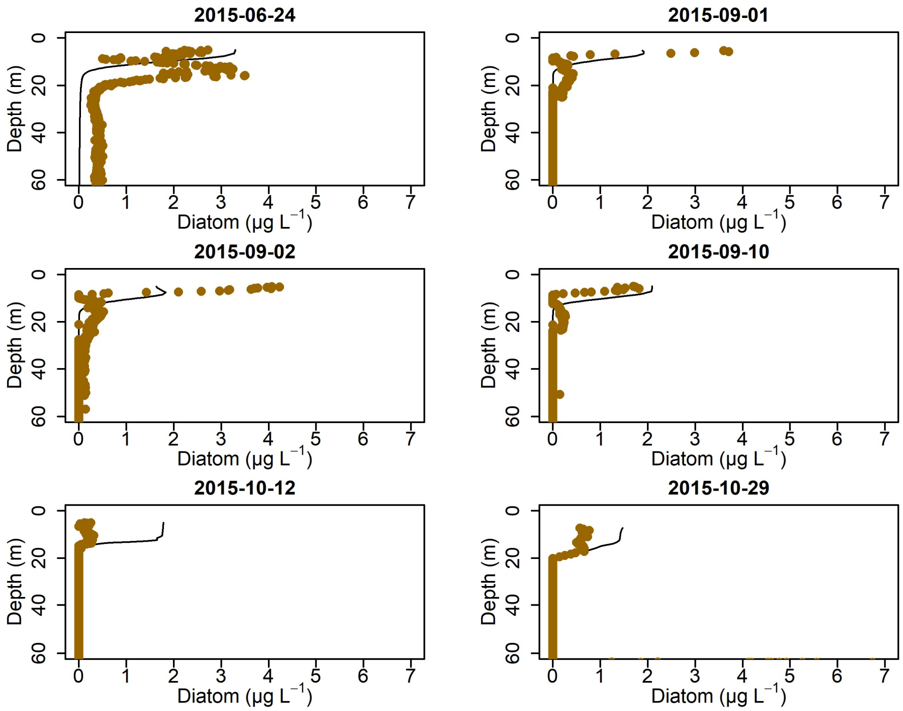


Fig. S7. The same as Fig. S6 but for the validation period.

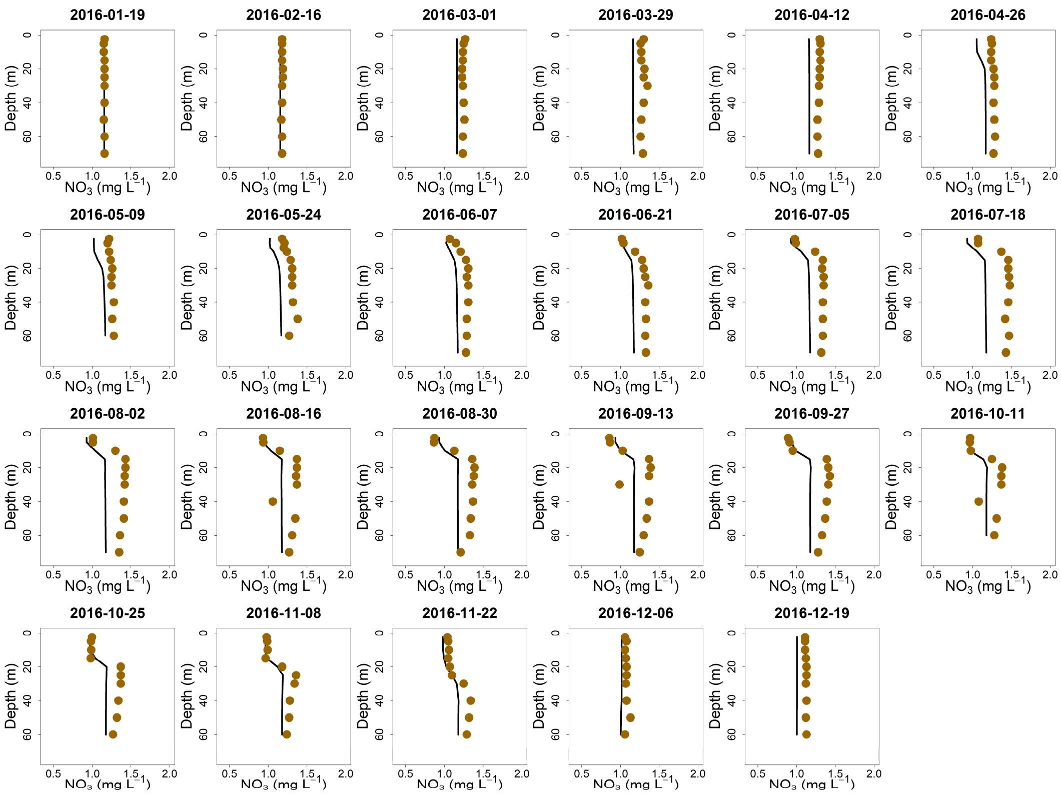


Fig. S8. Comparison between simulated (lines) and measured (points) nitrate during the calibration period.

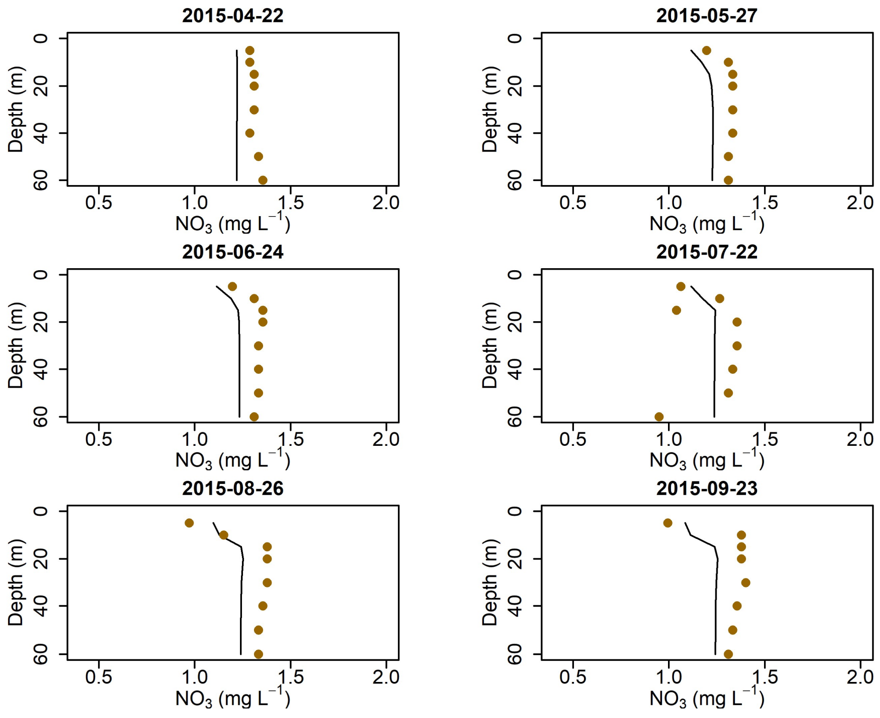


Fig. S9. The same as Fig. S8 but for the validation period.

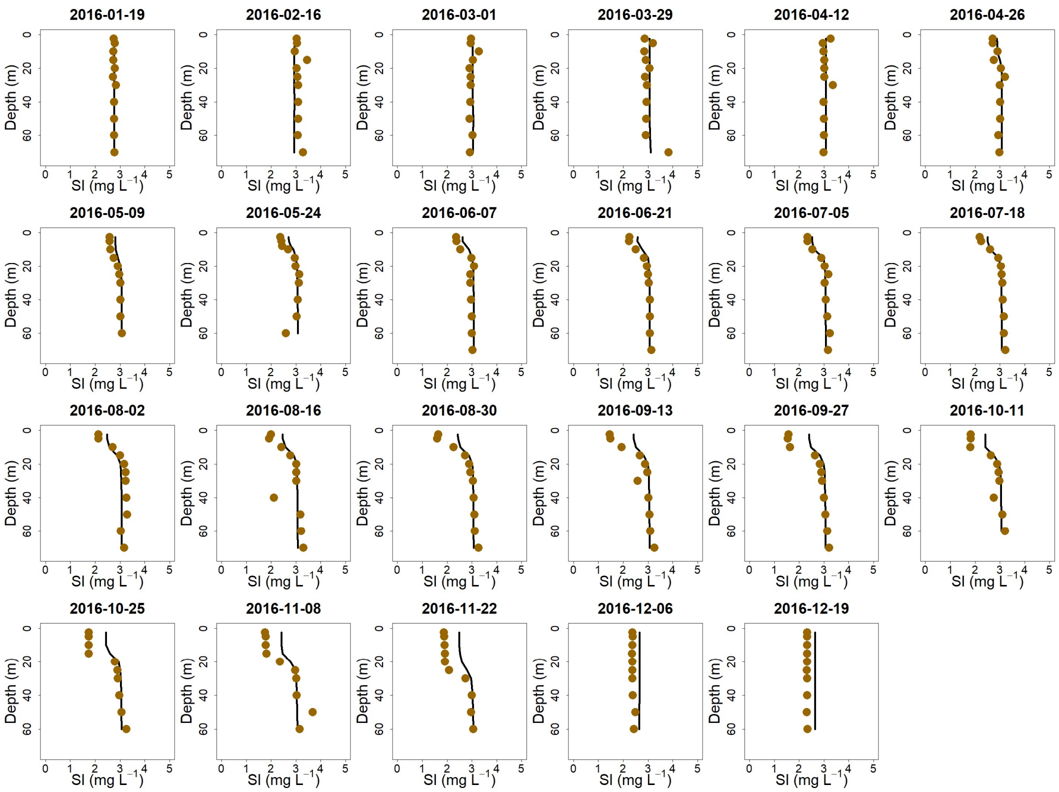


Fig. S10. Comparison between simulated (lines) and measured (points) silicate during the calibration period.

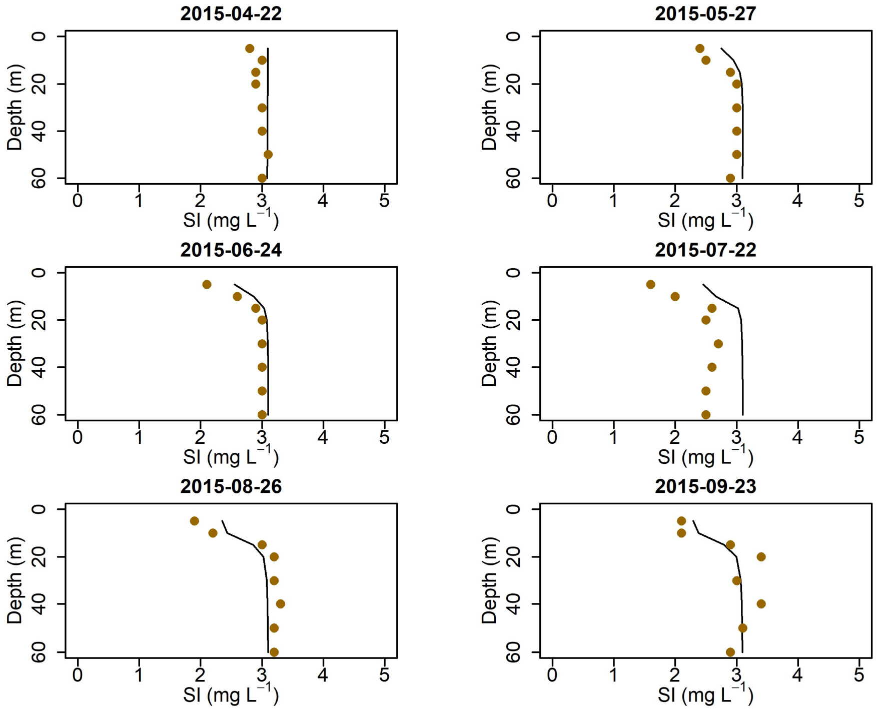


Fig. S11. The same as Fig. S10 but for the validation period.

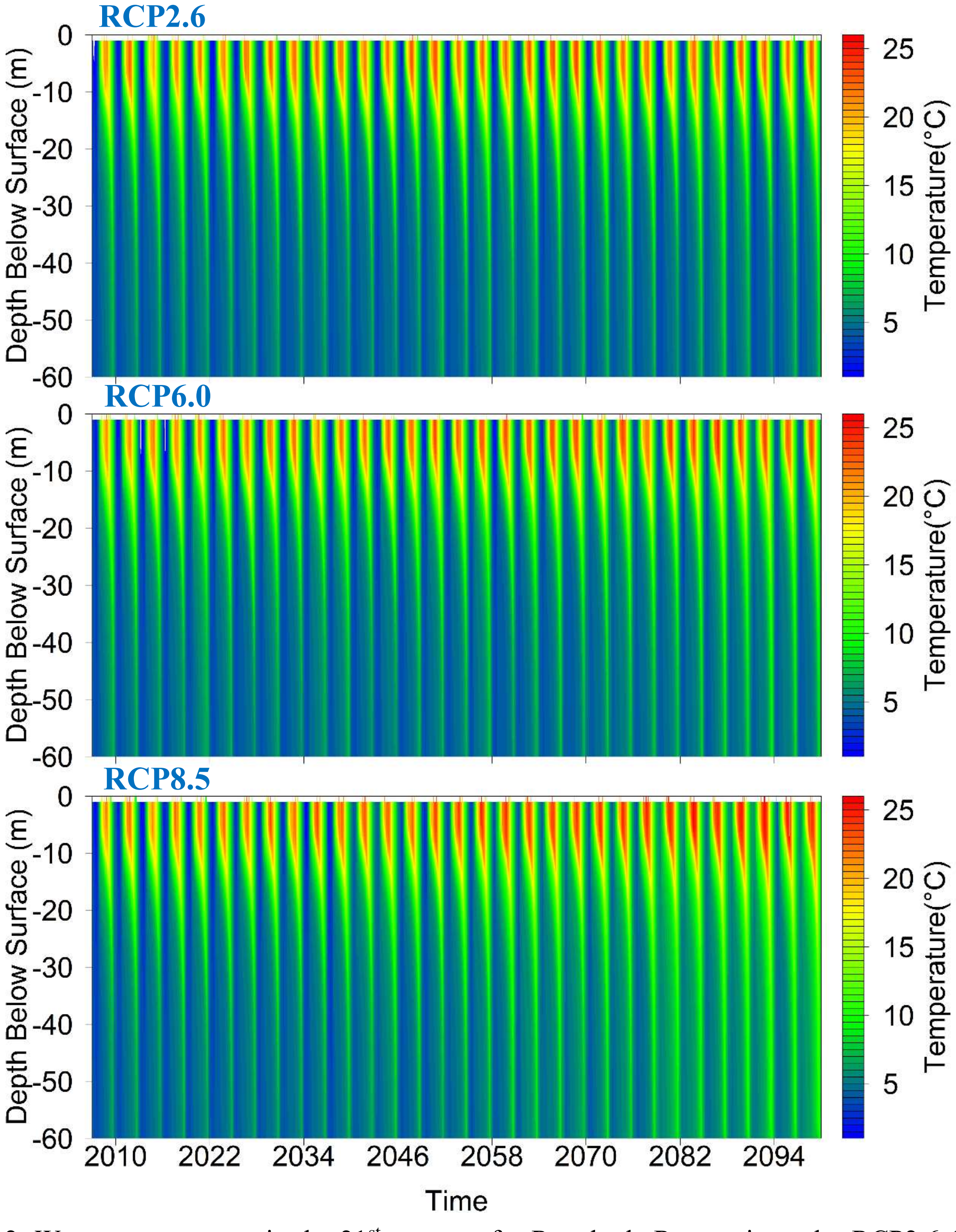


Fig. S12. Water temperatures in the 21st century, for Rappbode Reservoir, under RCP2.6 (top), RCP6.0 (middle) and RCP8.5 (bottom). In each subfigure it indicates the ensemble mean results driven by four climate models. To be noted, in each subfigure, it shows the discrete results of every 3 years between 2007 and 2097 (i.e. 2007, 2010, 2013…etc.), to better illustrate the changing pattern of water temperatures under future climate conditions.

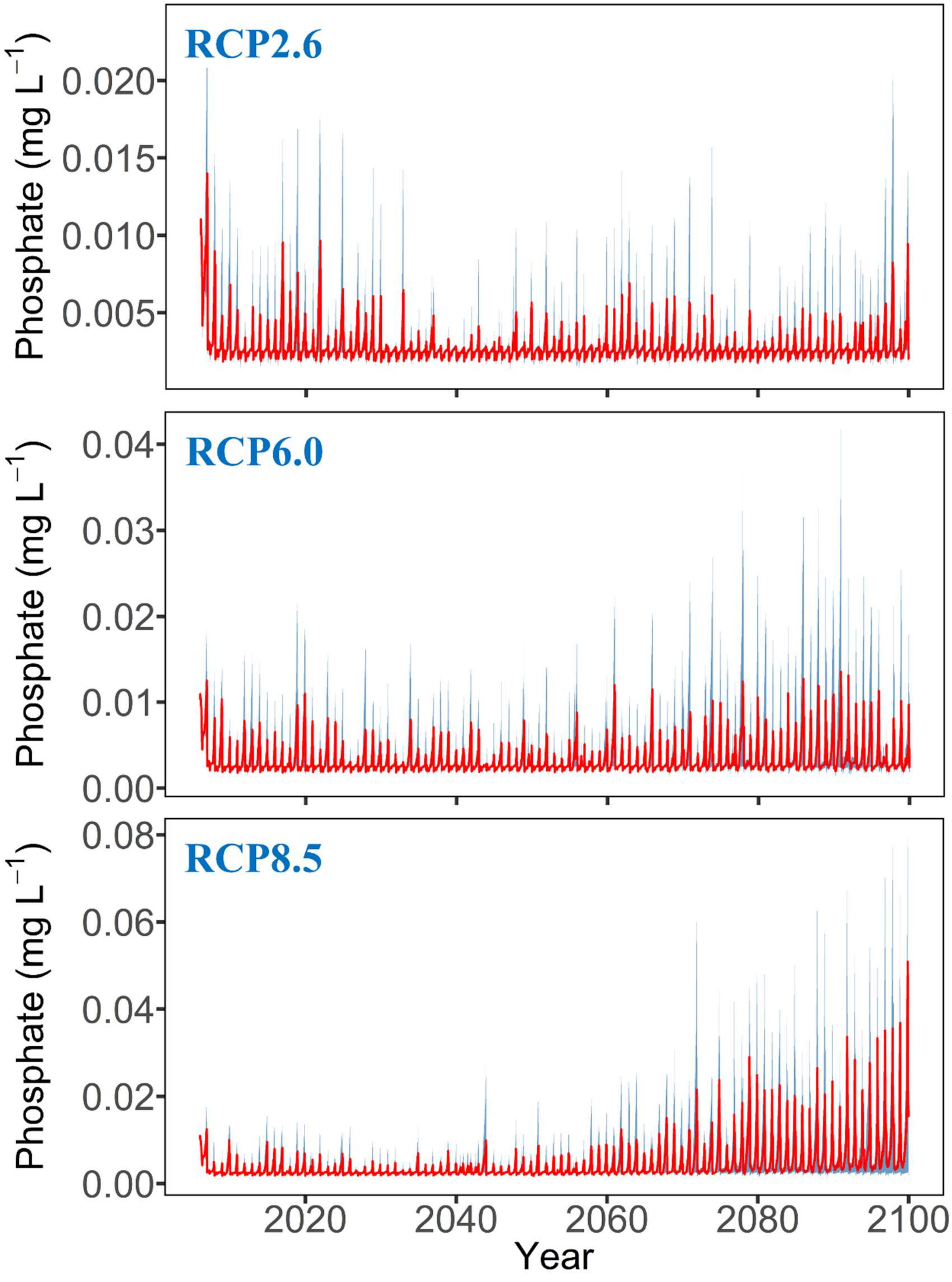


Fig. S13. Future projections of phosphate concentration, for Rappbode Reservoir, under RCP2.6 (upper), RCP6.0 (middle) and RCP8.5 (bottom). The red lines indicate the daily ensemble average results driven by four climate models, the blue shaded areas indicate the daily minimum and maximum results from the ensemble members.

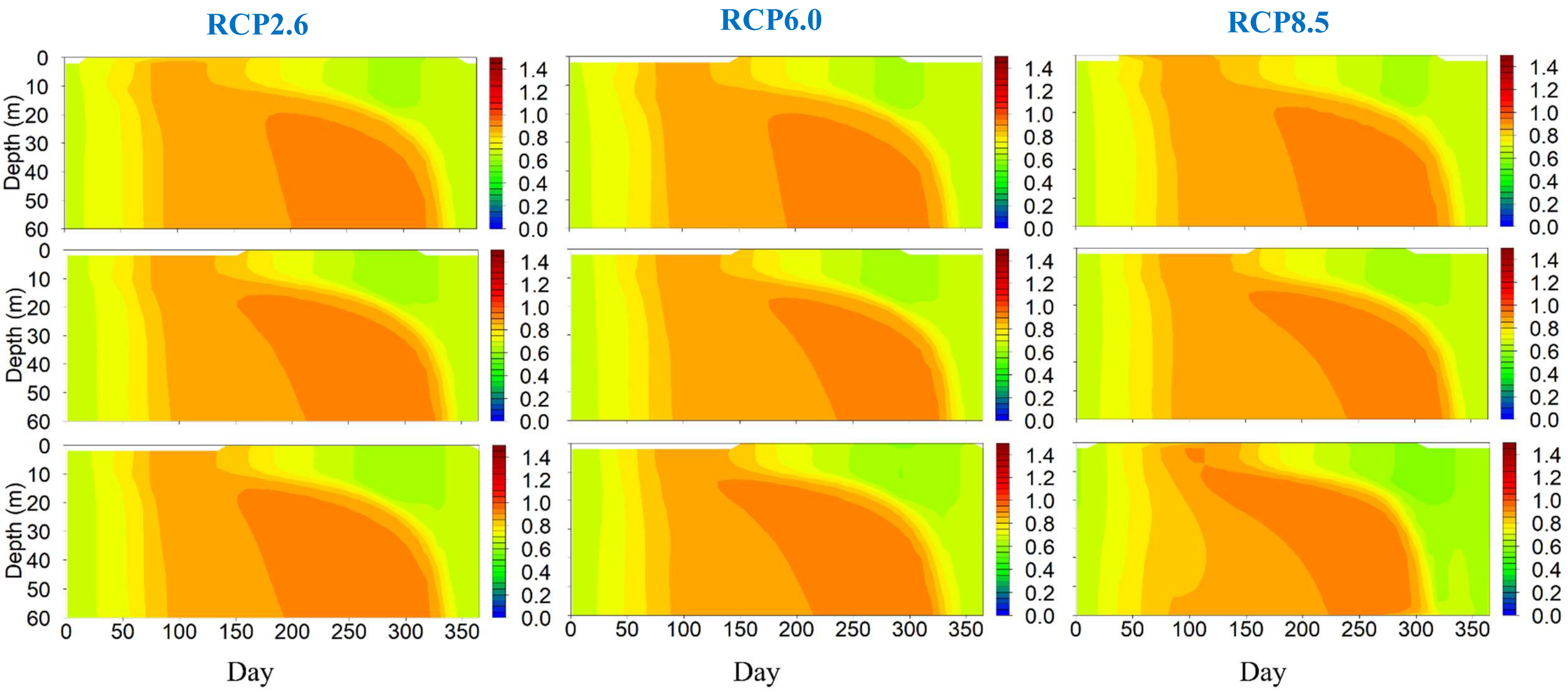


Fig. S14. Future projections of nitrate concentration (mg $L^{-1}$), for Rappbode Reservoir, under RCP2.6 (left), RCP6.0 (middle) and RCP8.5 (right). The top row indicates the ensemble average results, for every Julian day, in Period 07-21 (from 2007 to 2021), the middle row indicates the results in Period 45-59 (from 2045 to 2059) and the bottom row indicates the results in Period 85-99 (from 2085 to 2099).

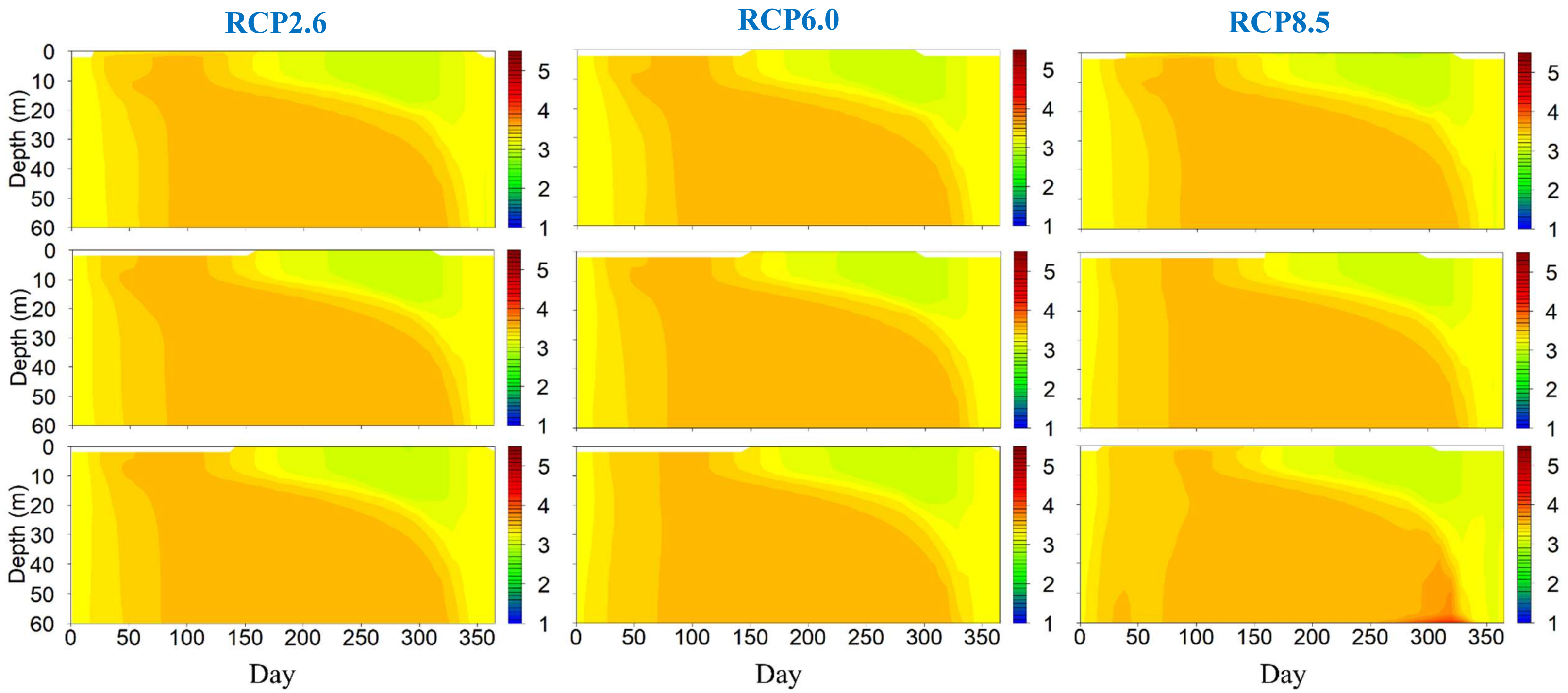


Fig. S15. The same as Fig. S14, but for silicate.

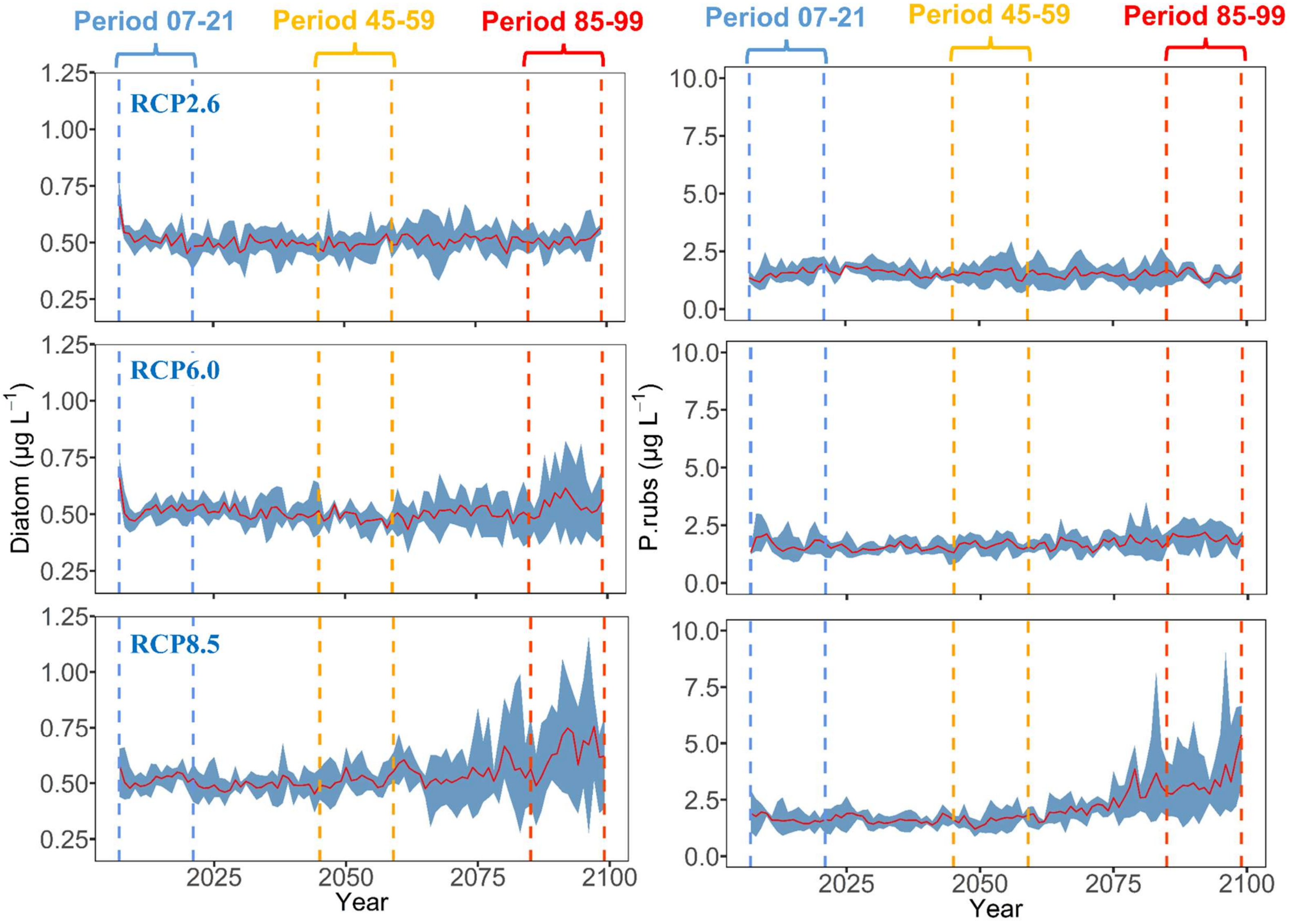


Fig. S16. Future projections of diatoms (left) and *P. rubescens* (right) concentration in the top 15 m, for Rappbode Reservoir, under RCP2.6 (upper), RCP6.0 (middle) and RCP8.5 (bottom). The red lines indicate the annual ensemble average results driven by four climate models, the blue shaded areas indicate the annual minimum and maximum results from the ensemble. Dashed lines, in the vertical direction, indicate 3 periods in the 21$^{st}$ century.

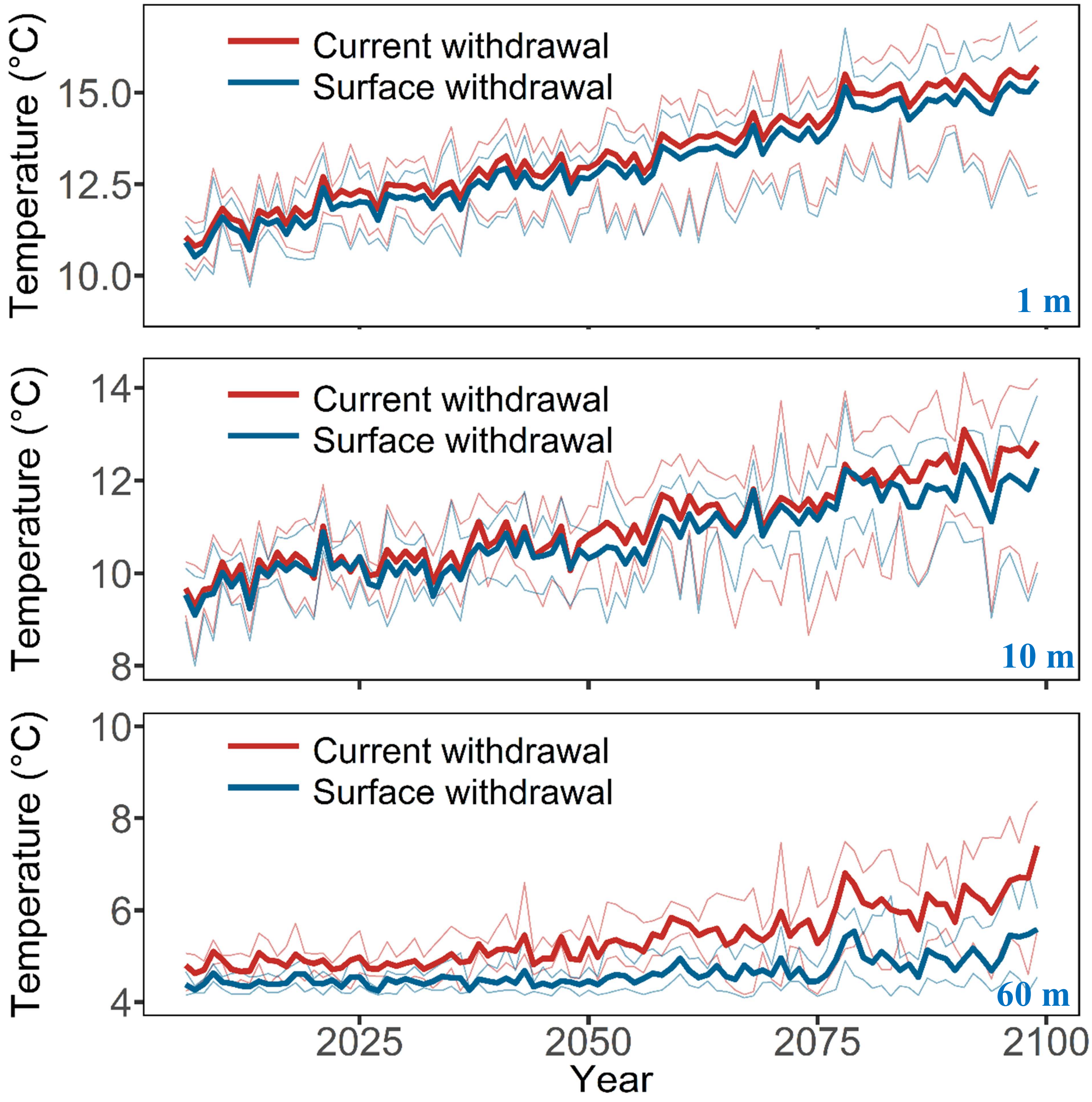


Fig. S17. Future projections of water temperature until the end of 21st century, for Rappbode Reservoir, at 1 m (top), 10 m (middle) and 60 m (below) driven by the current and surface withdrawal strategy under RCP8.5. The thick lines indicate the annual ensemble average results driven by four climate models, the thin lines indicate the annual minimum and maximum results from the ensemble.

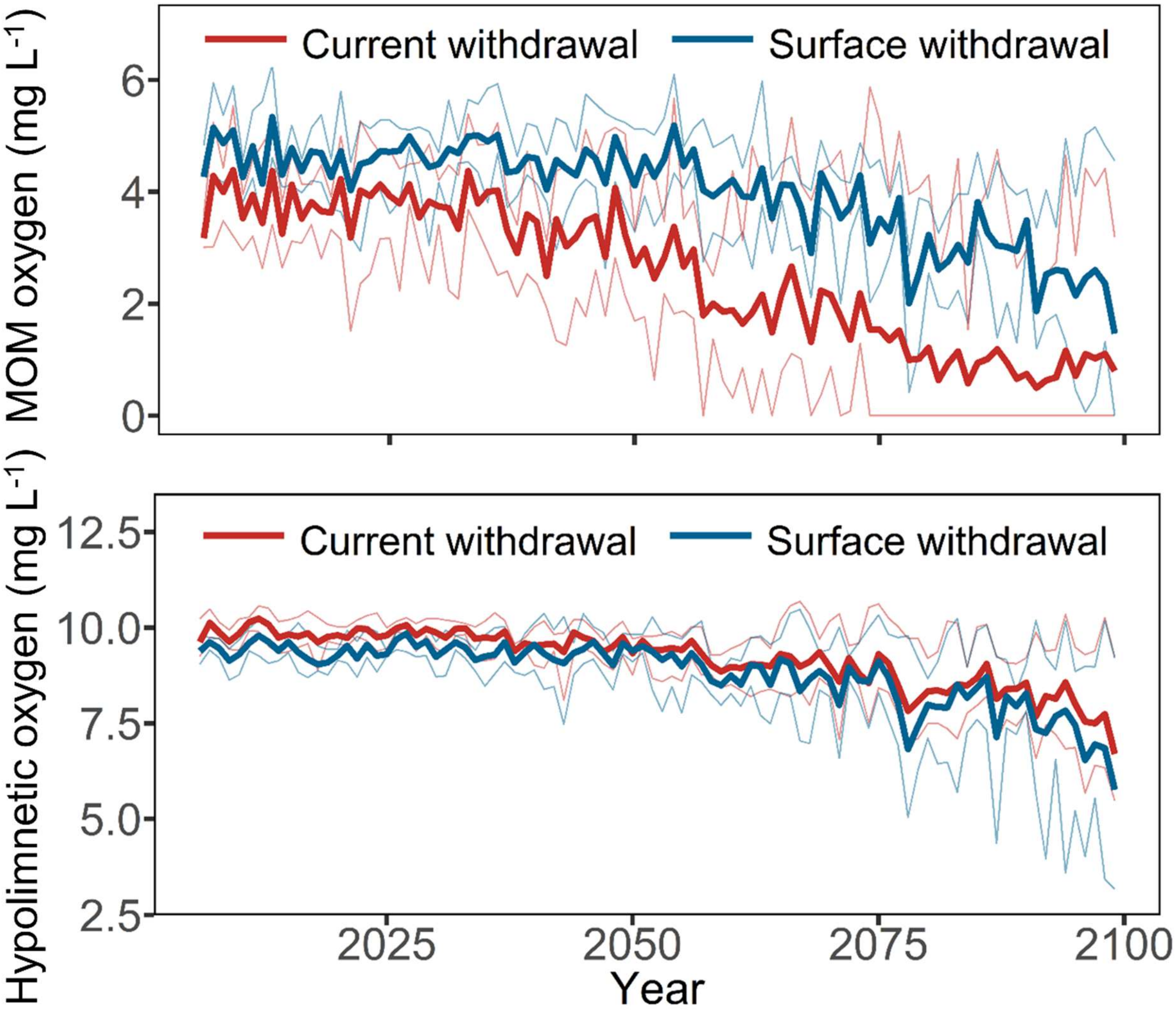


Fig. S18. Future projections of the MOM (metalimnetic oxygen minima) and hypolimnetic DO concentration, for Rappbode Reservoir, driven by the current and surface withdrawal strategy under RCP8.5. The thick lines indicate the annual ensemble average results driven by four climate models, the thin lines indicate the annual minimum and maximum results from the ensemble.

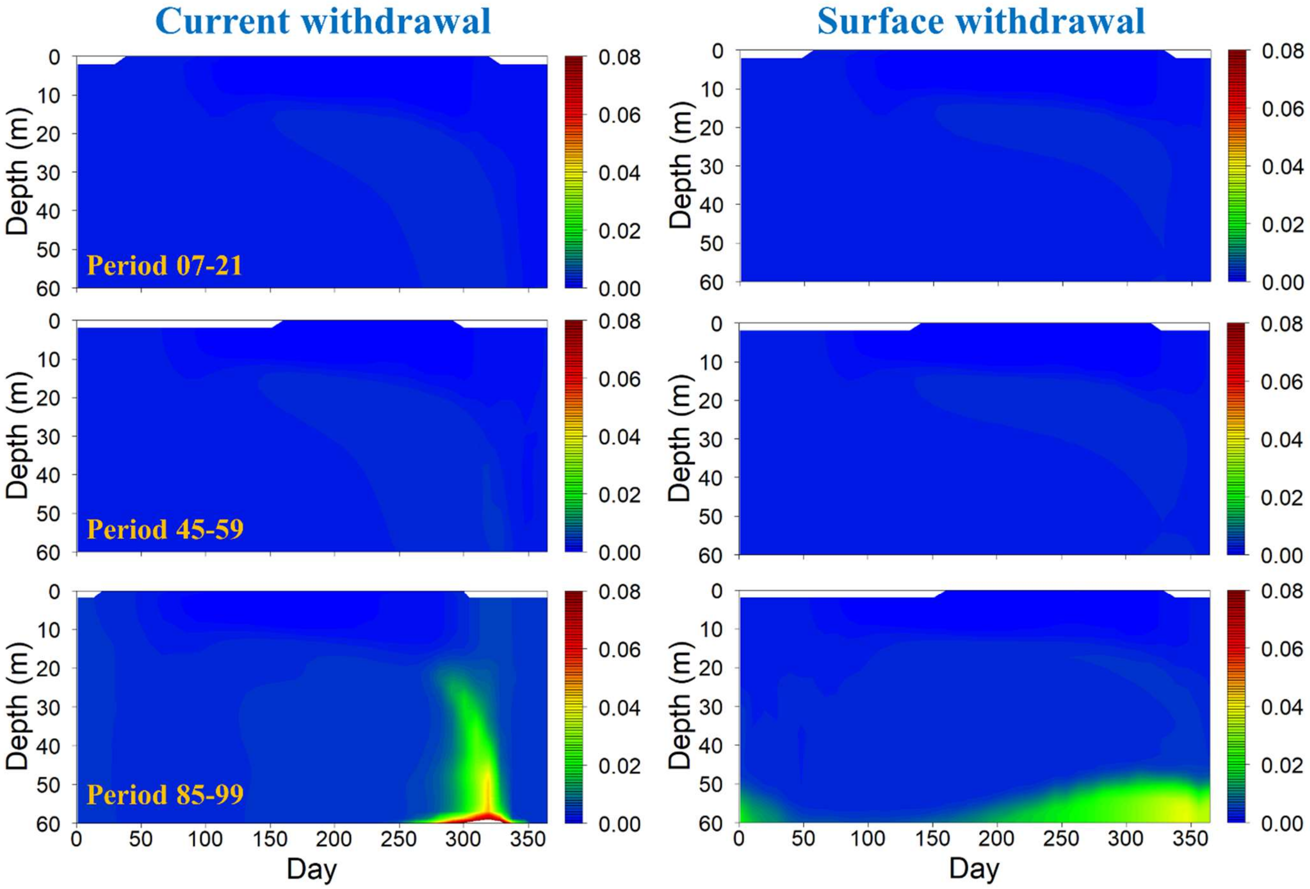


Fig. S19. Future projections of phosphate concentration (mg $L^{-1}$), for Rappbode Reservoir, driven by the current (left) and surface (right) withdrawal strategy under RCP8.5. The top row indicates the ensemble average results, for every Julian day, in Period 07-21 (from 2007 to 2021), the middle row indicates the results in Period 45-59 (from 2045 to 2059) and the bottom row indicates the results in Period 85-99 (from 2085 to 2099).

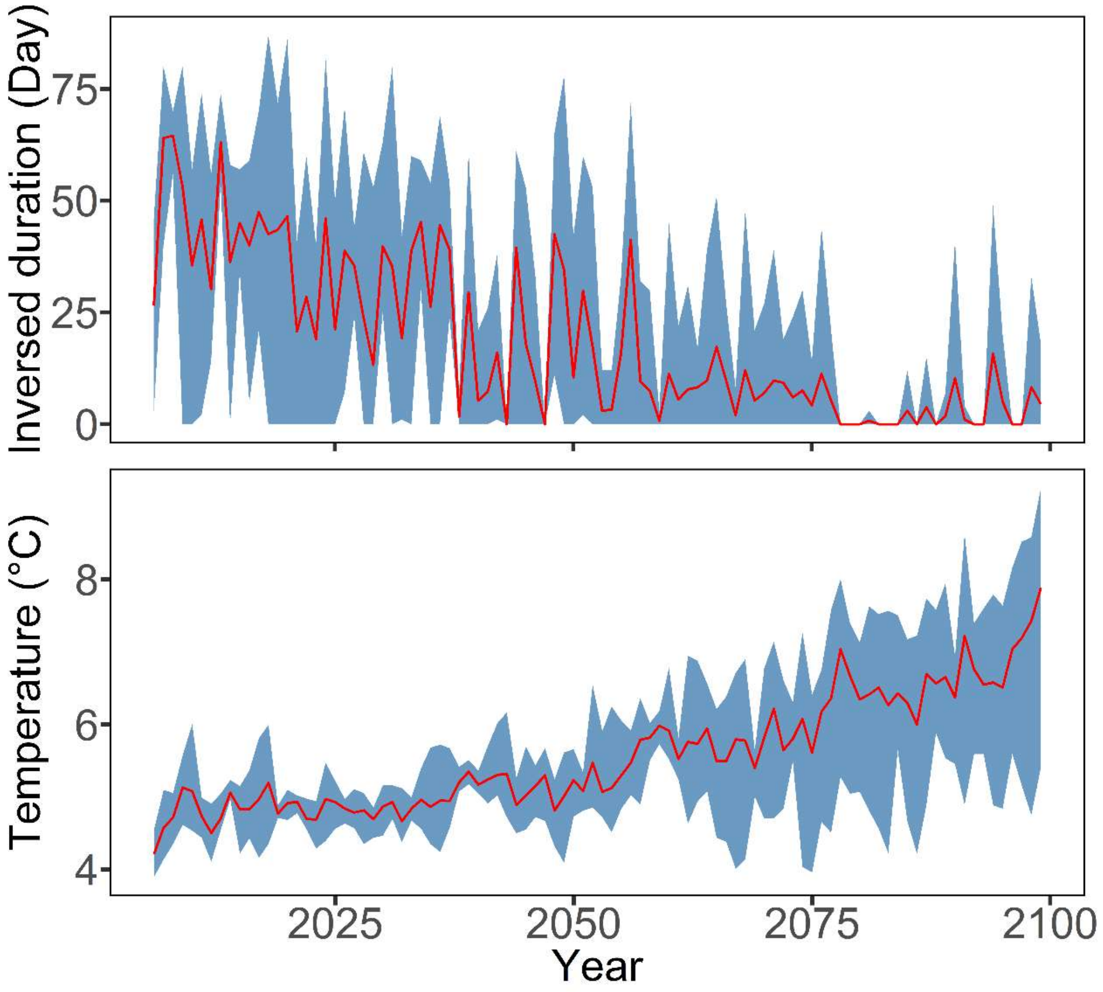


Fig. S20. Future projections for the duration of winter inversed stratification (top) and mixing temperature (bottom), for Rappbode Reservoir under RCP8.5. The red lines indicate the annual ensemble average results driven by four climate models, the blue shaded areas indicate the annual minimum and maximum results from the ensemble.

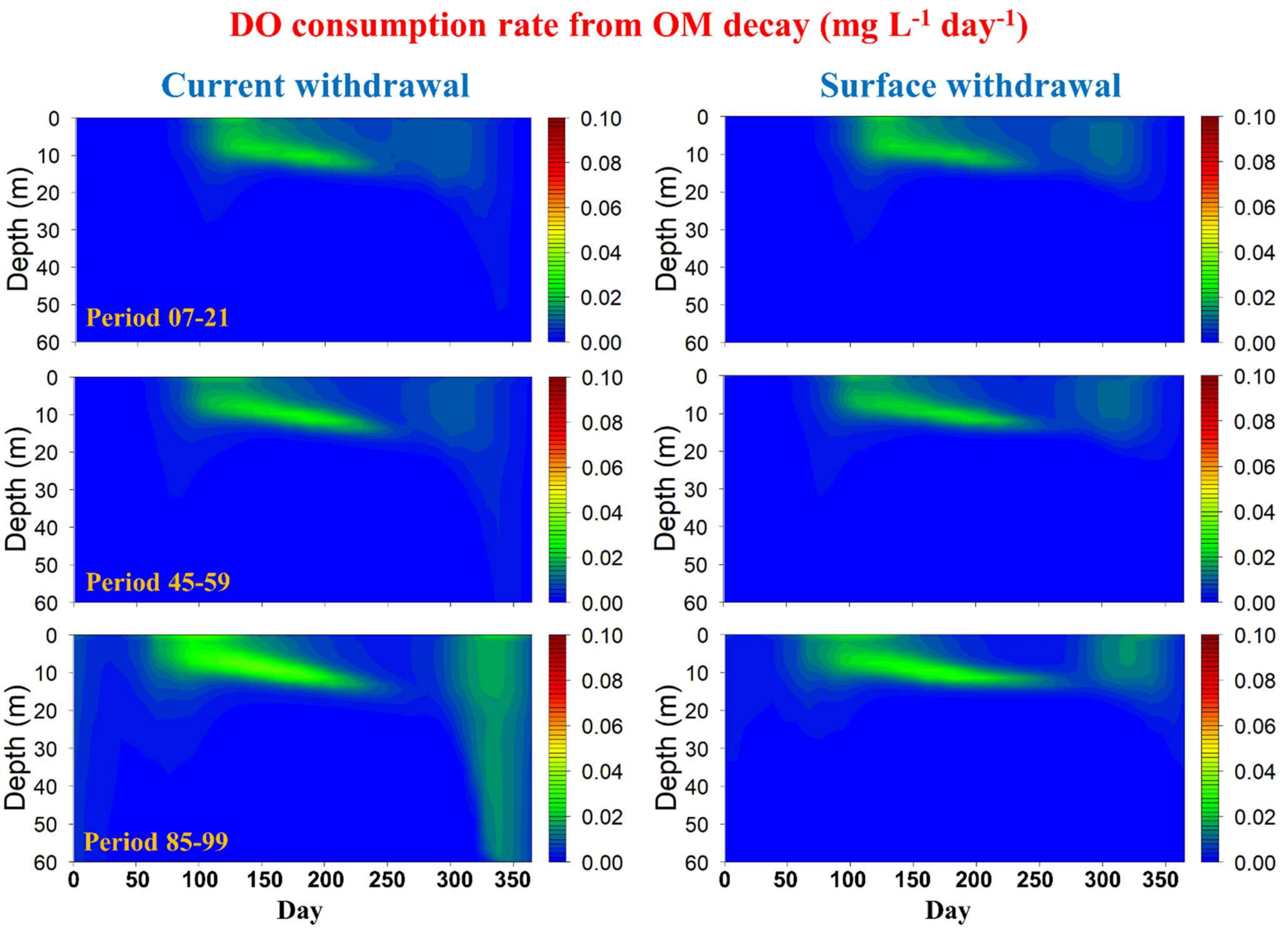


Fig. S21. DO consumption rate from organic matter (OM) decay driven by the current (left) and surface (right) withdrawal strategy under RCP8.5. The top row indicates the ensemble average results, for every Julian day, in Period 07-21 (from 2007 to 2021), the middle row indicates the results in Period 45-59 (from 2045 to 2059) and the bottom row indicates the results in Period 85-99 (from 2085 to 2099).

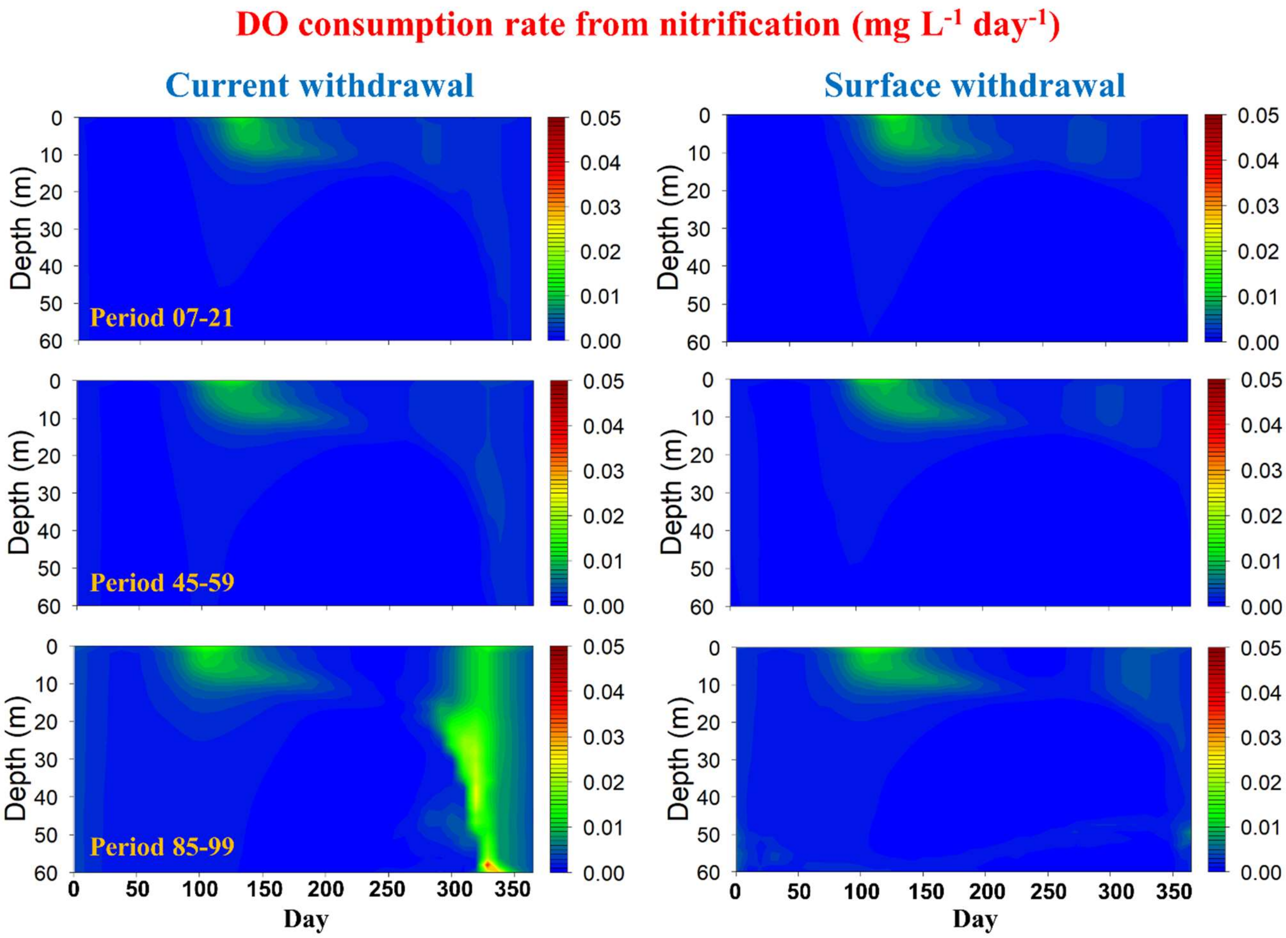


Fig. S22. The same as Fig.S21, but from nitrification.

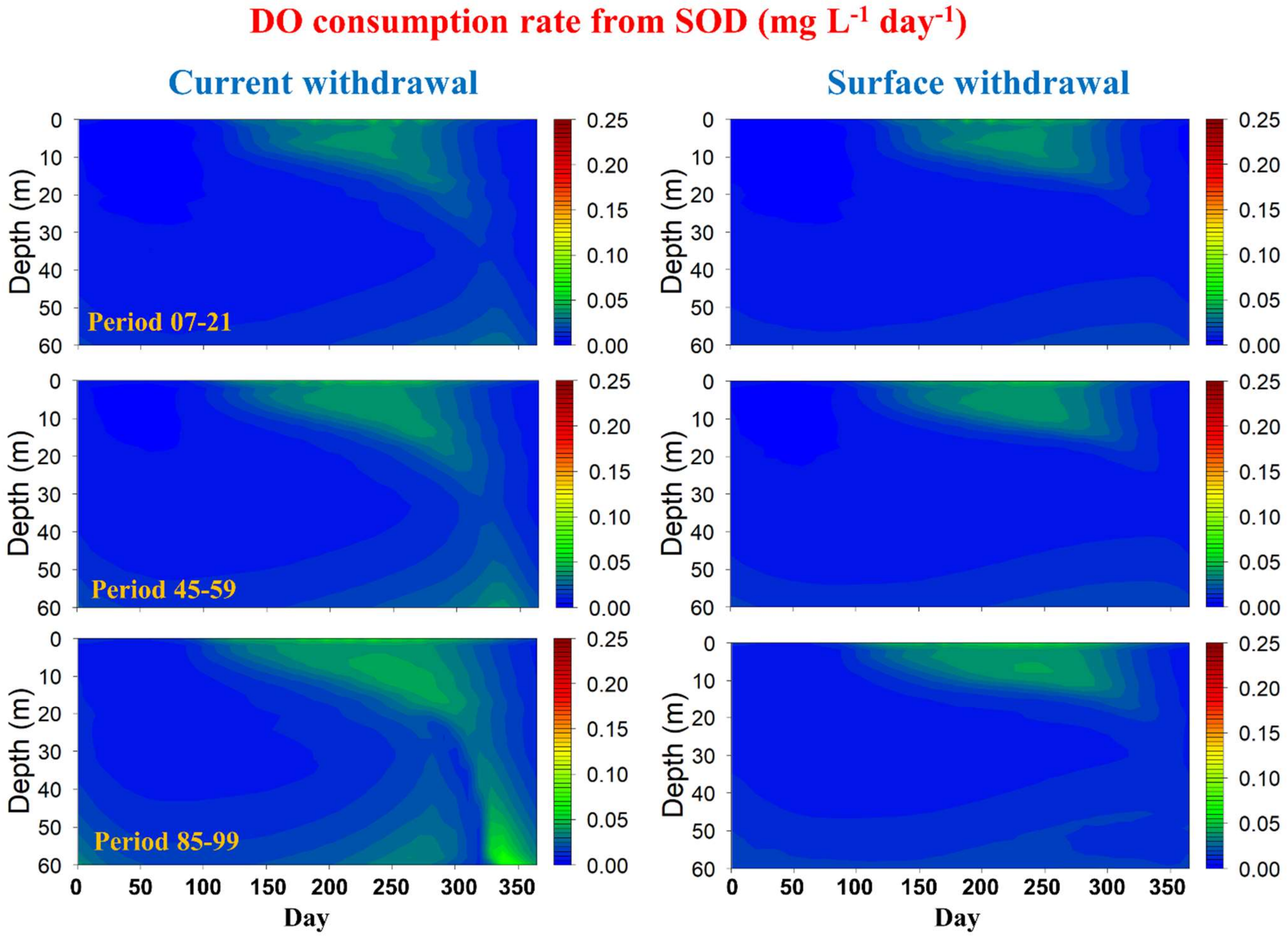


Fig. S23. The same as Fig.S21, but from SOD (sediment oxygen demand).

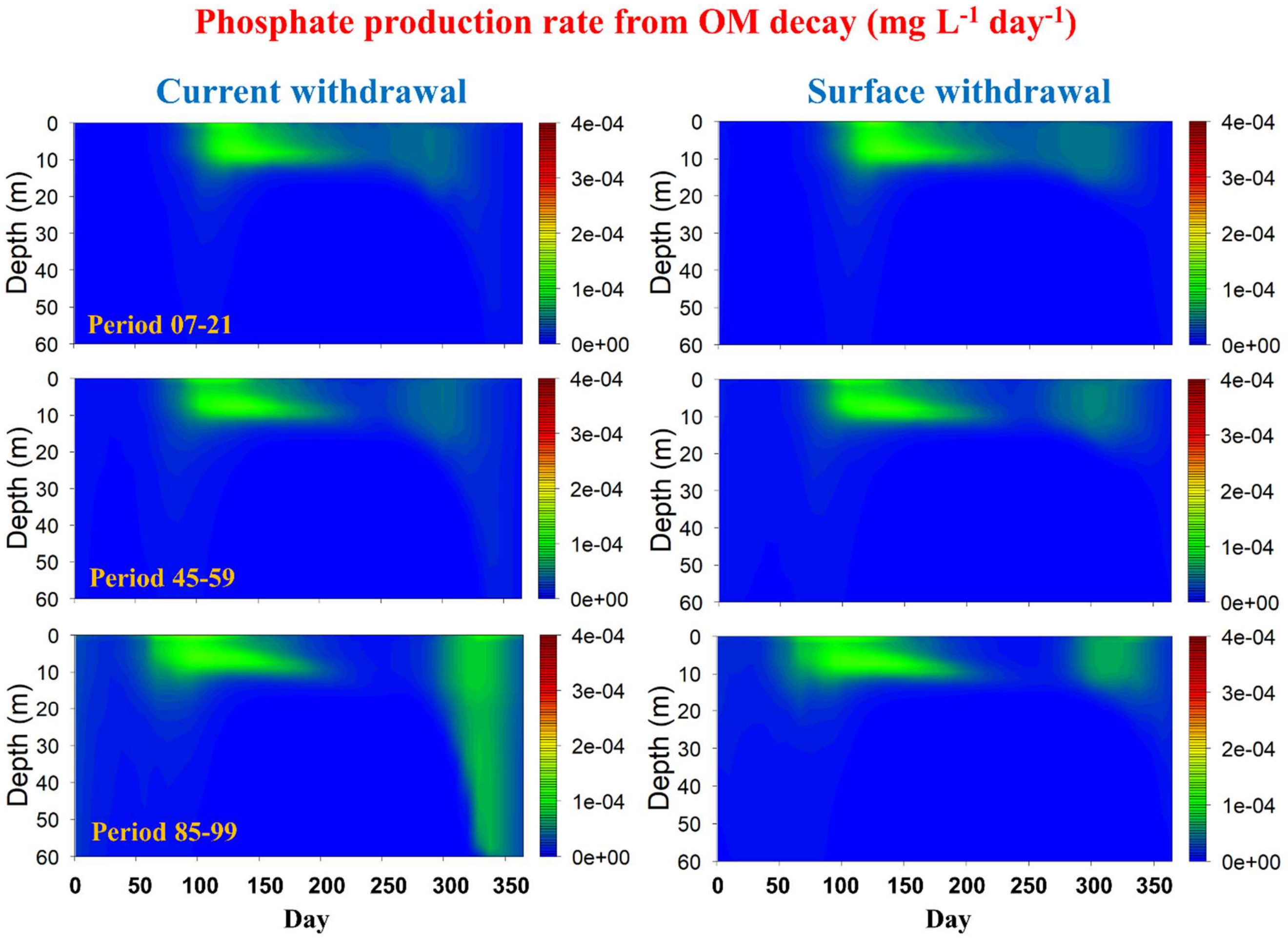


Fig. S24. Phosphate production rate from organic matter (OM) decay driven by the current (left) and surface (right) withdrawal strategy under RCP8.5. The top row indicates the ensemble average results, for every Julian day, in Period 07-21 (from 2007 to 2021), the middle row indicates the results in Period 45-59 (from 2045 to 2059) and the bottom row indicates the results in Period 85-99 (from 2085 to 2099).

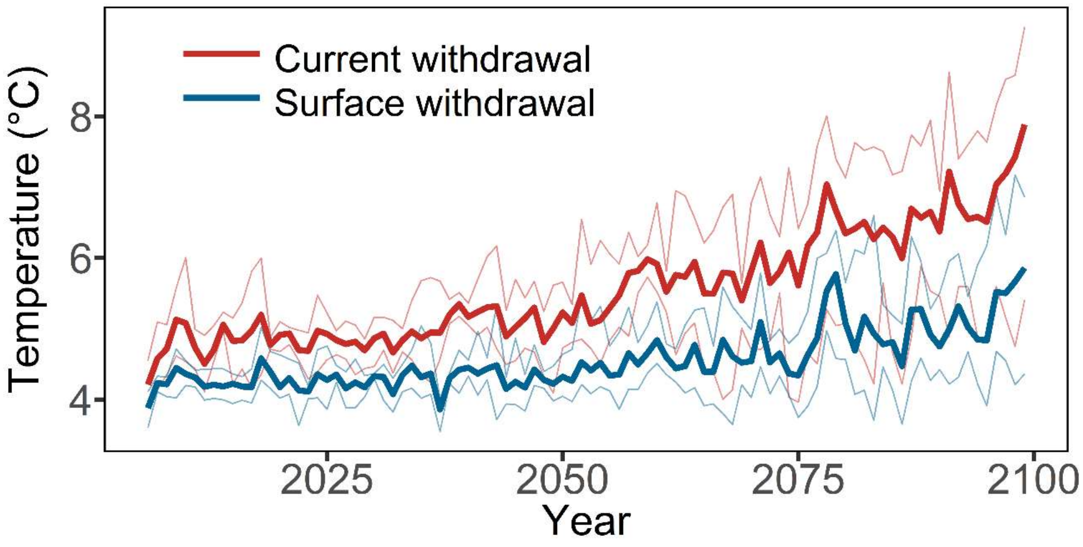


Fig. S25. Future projections for the mixing temperature, for Rappbode Reservoir driven by the current and surface withdrawal strategy under RCP 8.5. The thick lines indicate the annual ensemble average results driven by four climate models, the thin lines indicate the annual minimum and maximum results from the ensemble.

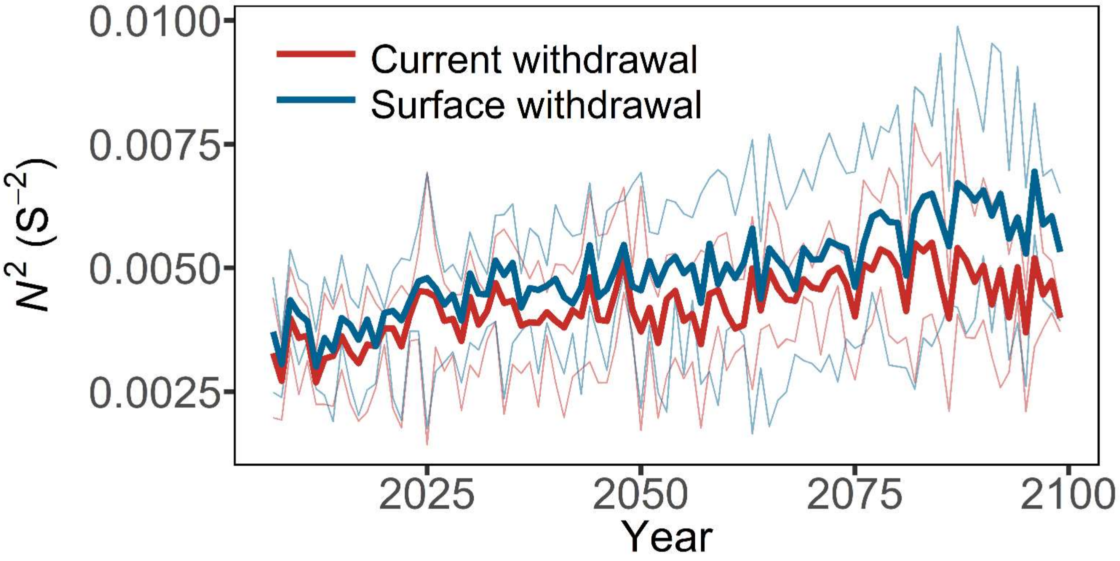


Fig. S26. Future projections of buoyancy frequency at the thermocline for Rappbode Reservoir, driven by the current and surface withdrawal strategy under RCP8.5 The thick lines indicate the annual ensemble average results driven by four climate models, the thin lines indicate the annual minimum and maximum results from the ensemble.

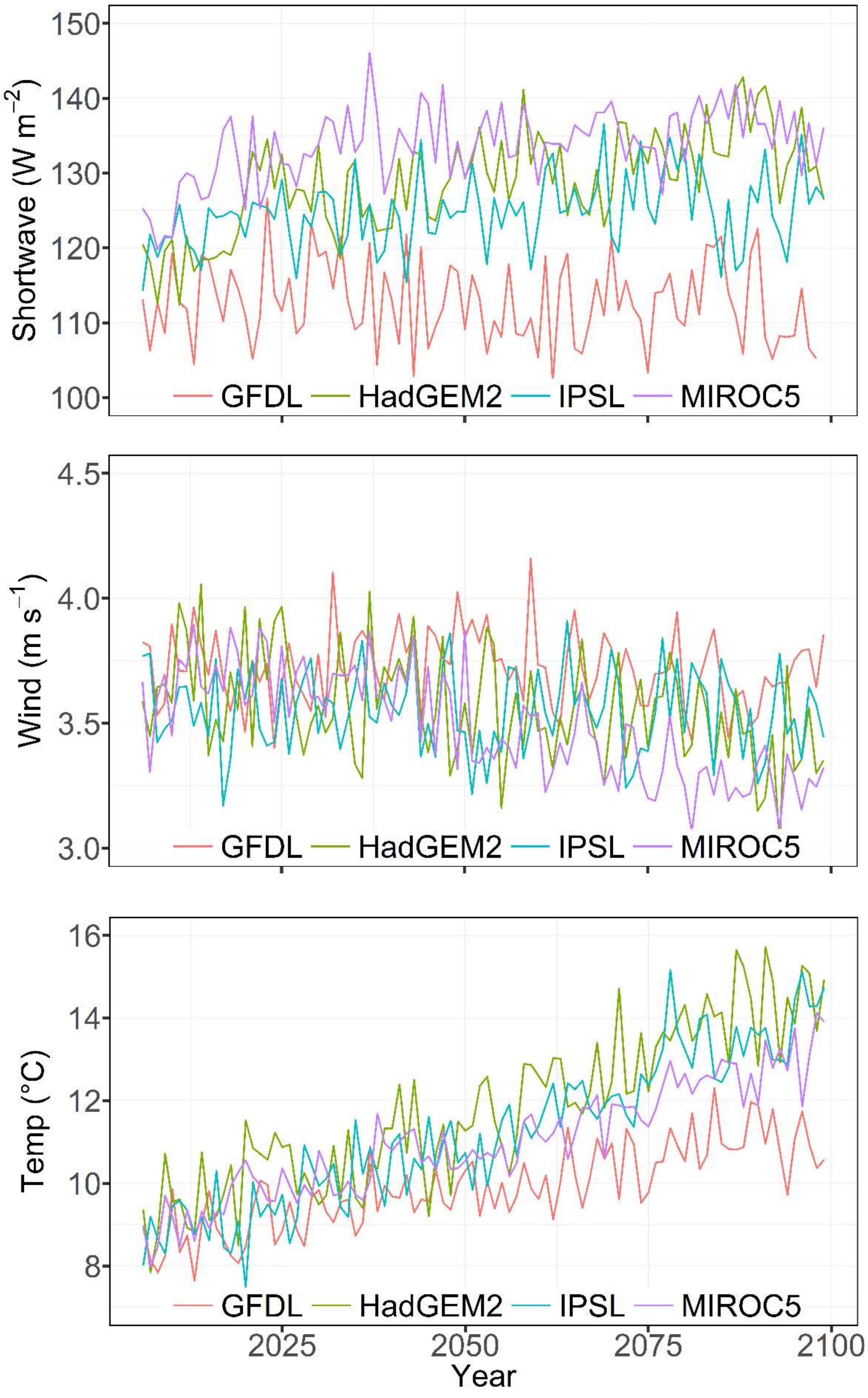


Fig. S27. Annual average projections of shortwave radiation (top), wind speed (middle) and air temperature (below), for Rappbode Reservoir, from four GCMs under RCP 8.5.

# Supplementary references


Boehrer, B. & M. Schultze, 2008. Stratification of lakes. Rev Geophy 46:RG 2005 doi:10.1029/2006RG000210.

Bowie, G. L., W. B. Mills, D. B. Porcella, C. L. Campbell, J. R. Pagenkopf, G. L. Rupp, K. M. Johnson, P. Chan, S. A. Gherini & C. E. Chamberlin, 1985. Rates, constants, and kinetics formulations in surface water quality modeling. EPA 600:3-85.

Brito, D., T. B. Ramos, M. C. Gonçalves, M. Morais & R. Neves, 2018. Integrated modelling for water quality management in a eutrophic reservoir in south-eastern Portugal. Environmental Earth Sciences 77(2):40.

Carraro, E., N. Guyennon, D. Hamilton, L. Valsecchi, E. C. Manfredi, G. Viviano, F. Salerno, G. Tartari & D. Copetti, 2012. Coupling high-resolution measurements to a three-dimensional lake model to assess the spatial and temporal dynamics of the cyanobacterium Planktothrix rubescens in a medium-sized lake Hydrobiologia. Springer, 77-95.

Chuo, M., J. Ma, D. Liu & Z. Yang, 2019. Effects of the impounding process during the flood season on algal blooms in Xiangxi Bay in the Three Gorges Reservoir, China. Ecological Modelling 392:236-249.

Cole, T. M. & S. A. Wells, 2006. CE-QUAL-W2: A two-dimensional, laterally averaged, hydrodynamic and water quality model, version 3.5.

Deliman, P. N. & J. A. Gerald, 2002. Application of the Two-Dimensional Hydrothermal and Water Quality Model, CE-QUAL-W2, to the Chesapeake Bay–Conowingo Reservoir. Lake Reserv Manage 18(1):10-19.

Dillencourt, M. B., D. M. Mount & N. S. Netanyahu, 1992. A randomized algorithm for slope selection. Int J Comput Geom Ap 2(01):1-27.

Feldbauer, J., R. Ladwig, J. P. Mesman, T. N. Moore, H. Zündorf, T. U. Berendonk & T. Petzoldt, 2022. Ensemble of models shows coherent response of a reservoir's stratification and ice cover to climate warming. Aquatic Sciences 84(4):50.

Fenocchi, A., M. Rogora, G. Morabito, A. Marchetto, S. Sibilla & C. Dresti, 2019. Applicability of a one-dimensional coupled ecological-hydrodynamic numerical model to future projections in a very deep large lake (Lake Maggiore, Northern Italy/Southern Switzerland). Ecological Modelling 392:38-51.

Kerimoglu, O., S. Jacquet, B. Vinçon-Leite, B. J. Lemaire, F. Rimet, F. Soulignac, D. Trévisan & O. Anneville, 2017. Modelling the plankton groups of the deep, peri-alpine Lake Bourget. Ecological Modelling 359:415-433.

Kobler, U. G., A. Wüest & M. Schmid, 2018. Effects of Lake–Reservoir Pumped-Storage Operations on Temperature and Water Quality. Sustainability 10(ARTICLE):1968 doi:https://doi.org/10.3390/su10061968.

Livingstone, D. M. & D. M. Imboden, 1996. The prediction of hypolimnetic oxygen profiles: a plea for a deductive approach. Canadian Journal of Fisheries and Aquatic Sciences 53(4):924-932.

Martynov, A., L. Sushama & R. Laprise, 2010. Simulation of temperate freezing lakes by one-dimensional lake models: performance assessment for interactive coupling with regional climate models.

McLeod, A. I., 2022. Kendall: Kendall Rank Correlation and Mann-Kendall Trend Test. R package version 2.2.1.

Mi, C., A. Sadeghian, K.-E. Lindenschmidt & K. Rinke, 2019. Variable withdrawal elevations as a management tool to counter the effects of climate warming in Germany's largest drinking water reservoir. Environmental Sciences Europe 31(1):19.

Mi, C., T. Shatwell, J. Ma, V. C. Wentzky, B. Boehrer, Y. Xu & K. Rinke, 2020. The formation of a metalimnetic oxygen minimum exemplifies how ecosystem dynamics shape biogeochemical processes: A modelling study. Water Research.

Muggeo, V. M. R., 2017. Interval estimation for the breakpoint in segmented regression: a smoothed score-based approach. Aust Nz J Stat 59(3):311-322.

Park, Y., K. H. Cho, J.-H. Kang, S. W. Lee & J. H. Kim, 2014. Developing a flow control strategy to reduce nutrient load in a reclaimed multi-reservoir system using a 2D hydrodynamic and water quality model. Science of the total environment 466:871-880.

Sadeghian, A., S. C. Chapra, J. Hudson, H. Wheater & K.-E. Lindenschmidt, 2018. Improving in-lake water quality modeling using variable chlorophyll a/algal biomass ratios. Environmental Modelling & Software 101:73-85.

Schlabing, D., M. A. Frassl, M. M. Eder, K. Rinke & A. Bárdossy, 2014. Use of a weather generator for simulating climate change effects on ecosystems: A case study on Lake Constance. Environmental modelling & software 61:326-338.

Shatwell, T., W. Thiery & G. Kirillin, 2019. Future projections of temperature and mixing regime of European temperate lakes. Hydrology and Earth System Sciences 23(3):1533-1551.

Smith, E. A., R. L. Kiesling, J. M. Galloway & J. R. Ziegeweid, 2014. Water quality and algal community dynamics of three deepwater lakes in Minnesota utilizing CE-QUAL-W2 models. US Geological Survey.

Tan, Z., H. Yao & Q. Zhuang, 2018. A small temperate lake in the 21st century: Dynamics of water temperature, ice phenology, dissolved oxygen and chlorophyll a. Water Resources Research.

Wentzky, V. C., M. A. Frassl, K. Rinke & B. Boehrer, 2019. Metalimnetic oxygen minimum and the presence of Planktothrix rubescens in a low-nutrient drinking water reservoir. Water Research(148):208-218.

Winslow, L., J. Read, R. Woolway, J. Brentrup, T. Leach, J. Zwart, S. Albers & D. Collinge, 2019. Package ‘rLakeAnalyzer’. Lake Physics Tools.

Wood, S. N., 2011. Fast stable restricted maximum likelihood and marginal likelihood estimation of semiparametric generalized linear models. Journal of the Royal Statistical Society Series B: Statistical Methodology 73(1):3-36.
